# Stepwise molecular mechanisms responsible for chemoresistance in bladder cancer cells

**DOI:** 10.1038/s41420-022-01242-8

**Published:** 2022-11-07

**Authors:** Jeong-Yeon Mun, Seung-Woo Baek, Mi-So Jeong, In-Hwan Jang, Se-Ra Lee, Jae-Young You, Jeong-Ah Kim, Gi-Eun Yang, Yung-Hyun Choi, Tae-Nam Kim, In-Sun Chu, Sun-Hee Leem

**Affiliations:** 1 Department of Biomedical Sciences, Dong-A University, Busan, 49315 Korea; 2 Korea Research Institute of Bioscience and Biotechnology (KRIBB), Daejeon, 34141 Korea; 3 Department of Bioinformatics, KRIBB School of Bioscience, Korea University of Science and Technology (UST), Daejeon, 34113 Korea; 4 Division of Drug Development & Optimization, Osong Medical Innovation Foundation (KBio), Daejeon, Chungbuk 28160 Korea; 5 Center for Scientific Instrumentation, Korea Basic Science Institute, Daejeon, Chungbuk 28119 Korea; 6 Department of Health Sciences, The Graduated of Dong-A University, Busan, 49315 Korea; 7 Department of Biochemistry, College of Oriental Medicine, Anti-Aging Research Center, Dong-eui University, Busan, 47227 Korea; 8 Department of Urology, Medical Research Institute, Pusan National University Hospital, Busan, 49241 Korea

**Keywords:** Tumour biomarkers, Prognostic markers

## Abstract

Chemotherapy resistance is an obstacle to cancer therapy and is considered a major cause of recurrence. Thus, understanding the mechanisms of chemoresistance is critical to improving the prognosis of patients. Here, we have established a stepwise gemcitabine-resistant T24 bladder cancer cell line to understand the molecular mechanisms of chemoresistance within cancer cells. The characteristics of the stepwise chemoresistance cell line were divided into 4 phases (parental, early, intermediate, and late phases). These four phase cells showed increasingly aggressive phenotypes in vitro and in vivo experiments with increasing phases and revealed the molecular properties of the biological process from parent cells to phased gemcitabine-resistant cell line (GRC). Taken together, through the analysis of gene expression profile data, we have characterized gene set of each phase indicating the response to anticancer drug treatment. Specifically, we identified a multigene signature (23 genes including *GATA3*, *APOBEC3G*, *NT5E*, *MYC*, *STC1*, *FOXD1*, *SMAD9*) and developed a chemoresistance score consisting of that could predict eventual responsiveness to gemcitabine treatment. Our data will contribute to predicting chemoresistance and improving the prognosis of bladder cancer patients.

## Introduction

Bladder cancer (BC) is one of the most prevalent genitourinary tumors worldwide, with approximately 573,000 new cases reported [1]. Approximately 80% of BC patients are diagnosed with non-muscle-invasive bladder cancer (NMIBC) with a high five-year survival rate, while the remaining 20% are diagnosed with muscle-invasive bladder cancer (MIBC) [2]. Although the surgical operation has been utilized to BC, patients with NMIBC frequently experience disease recurrence, of which about 20% progress to MIBC [3, 4]. Chemotherapy is a promising treatment for improving the survival rate of BC patients. Gemcitabine is a deoxycytidine analog that requires active cell membrane transport and represents an anticancer effect on various types of solid cancers, such as bladder cancer [5]. Once inside the cell, the difluorinated pro-drug undergoes mono-, di-, and tri-phosphorylation before incorporation into DNA, where it causes masked chain termination [6]. However, the response rate of MIBC patients to gemcitabine treatment has been shown to be limited to less than 40% efficacy, and only a small number of patients have potential benefits [7, 8].

Gene or protein markers related to gemcitabine resistance have been reported in various carcinomas [9, 10]. Among them, high expression of genes related to drug outflow pathways and DNA damage responses (DDR) has been reported alongside due to the secondary mutations in proteins or epigenetic changes in genes [11, 12]. There is an activation of the epithelial-mesenchymal transition (EMT) process, which may be interpreted as a strategy to avoid chemotherapy [13–15]. The studies of the chemical resistance mechanisms that have been mainly identified so far have focused on the mechanism that reflects the phenotype of the final resistance acquisition through comparison of the difference between the existing cancer cell lines and the resistance-acquired cell lines [16, 17]. Therefore, in order to better understand anticancer drug resistance to the characteristic of cancer with cell heterogeneity, it is necessary to understand the stepwise changes in the mechanism of chemical resistance.

In this study, the gemcitabine-resistance cancer (GRC) cell lines of BC were constructed into four time-points (parental, early, intermediate, and late phases) and their characteristics were analyzed focusing on understanding the mechanism by which cancer cells sequentially acquire resistance to chemotherapy. Cell lines constructed according to these four time-points were used to confirm cytological characteristics such as proliferation, metastasis, resistance, and gene expression profile data (RNA-seq). The data were generated to provide an opportunity to investigate the molecular mechanisms of stepwise-changing cell lines. As for the result, multiple markers explained these stepwise changes and screened to illustrate the complex mechanisms of chemoresistance through bioinformatics analysis. Taken together, 23-gene signatures were identified as stepwise chemoresistance markers, where chemoresistance score was developed to predict reactivity to the gemcitabine treatment, and its potential usefulness was verified in the clinical cohort of BC.

## Results

### Construction of sequential gemcitabine-resistant-bladder cancer cell lines

**Fig. 1:**
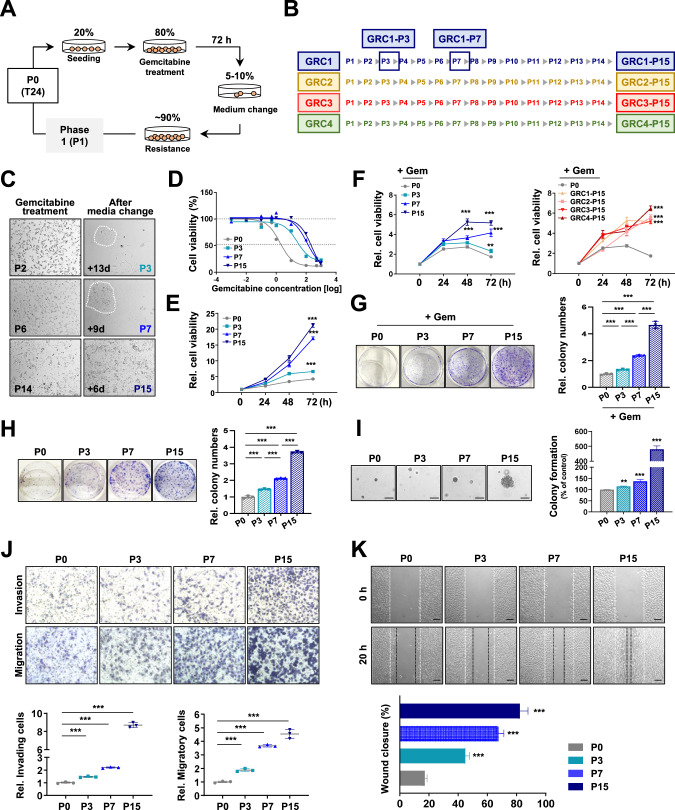
Establishment and characterization of sequential gemcitabine-resistant cancer (GRC) cell lines. **A** Schematic overview showing the establishment of sequential GRC cell lines. **B** Four types of the GRC cell lines (GRC1, GRC2, GRC3, and GRC4) were constructed from P1 to P15. Early phase P3, intermediate phase P7, and late phase P15 of the GRC1 cell line were used as sequential GRC models (blue squares). **C** Changes in the GRC1 cell line when the phase increases by one step after gemcitabine treatment. The first two pictures show the elapsed time (d, day) and colony morphology during proliferation from P2 to P3, the middle pictures from P6 to P7, and the last pictures from P14 to P15. **D** Gemcitabine sensitivity curves for each concentration of the GRC1 cell line. Various gemcitabine concentrations [μM] were obtained by MTT analysis after 72 h of treatment. The average of the experimental values repeated at least three times are shown. **E** Cell survival assays were performed to determine the cell viability of early phase P3, intermediate phase P7, and late phase P15 compared to parental phase P0 at 0, 24, 48, and 72 h. **F** Cell survival assays were performed in the late phase P15 of GRC2-4 cell lines and GRC1 cell line compared to parental P0 with 300 nM of gemcitabine treatment condition. **G** Anchorage-dependent growth assay was performed in the GRC1 cell line with treated 300 nM of gemcitabine. **H**, **I** Anchorage-dependent (**H**) and anchorage-independent (**I**) growth assays were performed in the GRC1 cell line and parental phase P0 for 7 days. **J** Cell invasion and migration abilities of the GRC1 cell line were enhanced compared to parental phase P0. Cells were detected and calculated by counting cells per field. ×400. **K** A wound-healing assay was performed to examine the ability of wound closure of the GRC1 cell line compared to parental phase P0. Monolayers were scratched using a 200 μl pipette tip and then photographed at 0 and 20 h. The extent of wound healing was quantified using ImageJ software and the percentage of wound closure was calculated. **P < 0.01; ***P < 0.001.

Chemotherapy resistance is the biggest obstacle in many cancer patients, and this is closely related to the survival rate due to treatment failure or recurrence [8]. In general, the mechanism of drug-specific resistance was studied by simple comparison with cancer cells that obtained resistance. In this study, we focused on the molecular mechanisms that cancer cells acquire through gemcitabine exposure in the T24 bladder cancer cell line. We have illustrated a workflow diagram for analysis (Fig. S1). By defining one phase as confirming the survival of the cell line we sequentially constructed a total of 15 phases with GRC cell lines (Fig. 1A). In addition, a total of four different types (GRC1, GRC2, GRC3, and GRC4) of GRC cell lines and four time-points (parental phase P0, early phase P3, intermediate phase P7, and late phase P15) were defined by the method schematized (Fig. 1B). After gemcitabine treatment for 72 h and the medium was exchanged, we observed colonies formed faster and larger as the phase increased in the GRC1 cell line (Fig. 1C). In addition, it was confirmed that cell viability was fully restored in late phase P15 of the other three GRC cell lines (GRC2-P15, GRC3-P15, and GRC4-P15, Fig. S2A). In all phases of the GRC cell lines, drug sensitivity and cell viability were significantly improved compared to parental phase P0 (Fig. 1D, E and Fig. S2B, C). Late phase P15 of the GRC cell lines with gemcitabine treatment showed significantly increased cell viability and colony formation activity compared with parental phase P0 (Fig. 1F, and Fig, S2D). As well, all phases of GRC1 cell line with the treatment of gemcitabine increased cell viability and colony formation activity (Fig. 1F, G). The cell viability of the GRC1 cell line increased according to the rise in phases, especially in early phase P3, intermediate phase P7, and late phase P15 (Fig. S2E). We investigated the anchorage-dependent and independent-growth assays in early phase P3, intermediate phase P7, and late phase P15 of the GRC1 cell line, and confirmed a stepwise increase in the colony formation ability and anchorage-independent-growth activity (Fig. 1H, I). Through cell migration, invasion, and wound-healing assays for the GRC cell lines, it was confirmed that early phase P3, intermediate phase P7, and late phase P15 were significantly facilitated than in parental phase P0 (Fig. 1J, K). The other GRC2-4 cell lines of late phase P15, similarly, confirmed an increasing tendency compared to parental phase P0 (Fig. S2H–K). The other GRC cell lines were also confirmed to be stable through subculture, and subsequent experiments and analyzes were performed at four time-point of the GRC1 cell line.

### Characterization of genes related to the EMT process in sequential gemcitabine-resistant-bladder cancer cell line

**Fig. 2:**
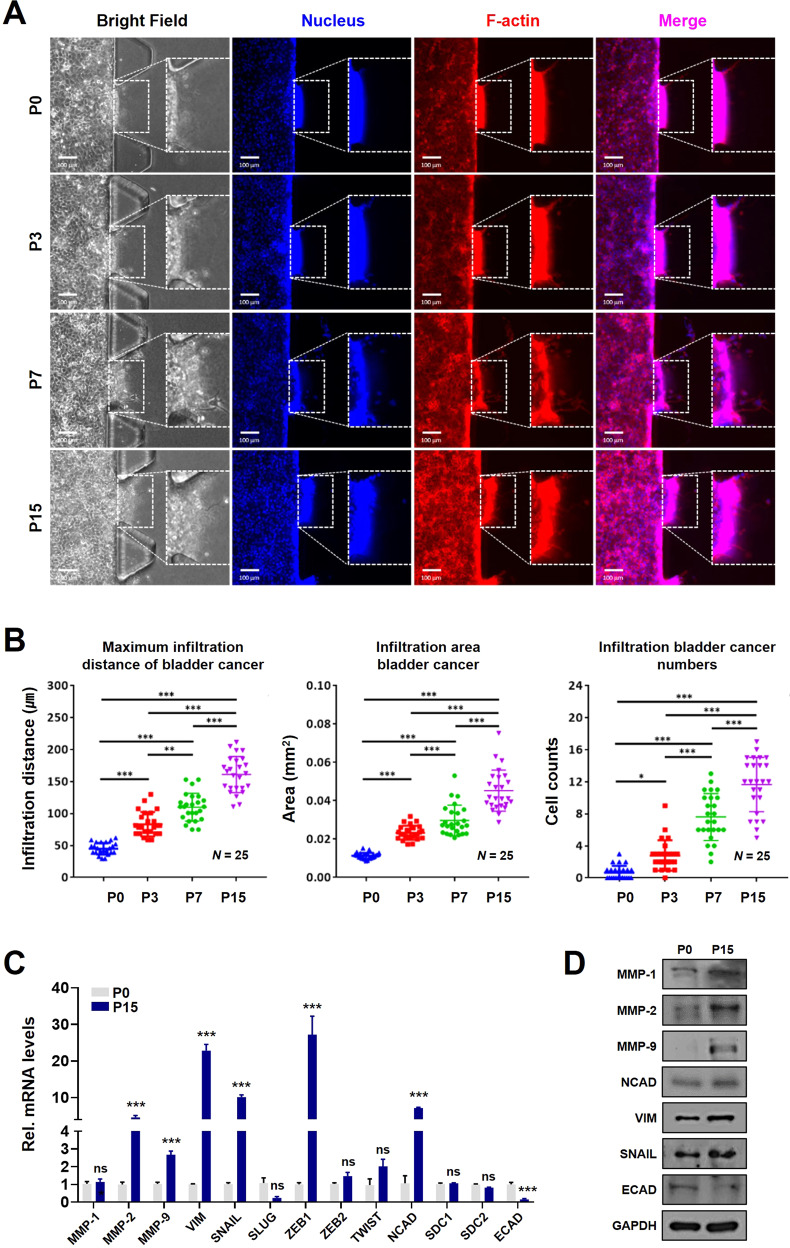
Cell mobility in 3D conditions and expression of EMT-related genes in the GRC1 cell line. **A** Representative image of the GRC1 cell line invading the gel on a microfluidic device. **B** The results of quantitative analysis of the maximum infiltration distance, infiltration area, and infiltration numbers of the GRC1 cell line using ImageJ software. **C** Confirmation of the mRNA and **D** protein levels of EMT-related genes in parental phase P0 and late phase P15. ns not significant; *P < 0.05; ***P < 0.001.

A fatal problem for patients who are resistant to chemotherapy is the increase in malignancy and metastasis of cancer, which is considered to be the leading cause of cancer-related death. Several recent studies have reported that EMT plays an important role in chemotherapy resistance and contributes to cancer metastasis and recurrence after chemotherapy [13–15]. The three-dimensional (3D) cell culture system is a major technique for confirming the behavior of cancer cells, and through the study of the 3D culture system, cell proliferation, cell-to-cell signaling, metastasis, cell migration, invasion, and angiogenesis can be confirmed [18, 19]. Cell mobility previously confirmed in the 2D culture conditions was reconfirmed by culturing the cell lines in a microfluidic device to confirm the mobility of the GRC1 cell line under 3D conditions (Fig. 2A). After incubating the GRC1 cell line at each phase in microfluidic devices, cell mobility was confirmed through the nucleus and F-actin immunostaining. As a result, the number of cells infiltrated from the GRC1 cell line increased according to each phase compared to parental phase P0 (Fig. 2A). When the maximum infiltration distance, infiltration area, and the number of infiltrating cells for each phase were calculated, a significant increase was observed (Fig. 2B). Next, the expression level of EMT-related markers was investigated in the GRC1 cell line in which cell mobility was confirmed. Comparing parental phase P0 and late phase P15 in the GRC1 cell line, the mRNA expression levels of *MMP2, MMP9, VIM, SNAIL, ZEB1*, and *NCAD*, which were positive markers of EMT [20, 21], were significantly increased. Alternatively, the mRNA expression level of *ECAD*, an epithelial marker, was significantly decreased (Fig. 2C). In addition, the protein expression levels of MMP-1, -2, -9, NCAD, VIM, and SNAIL were increased, and ECAD was decreased in late phase P15 compared to parental phase P0 (Fig. 2D). These results suggest that there is a significant association between the acquisition of gemcitabine resistance and EMT.

### Time-point analysis of gene expression profile data

**Fig. 3:**
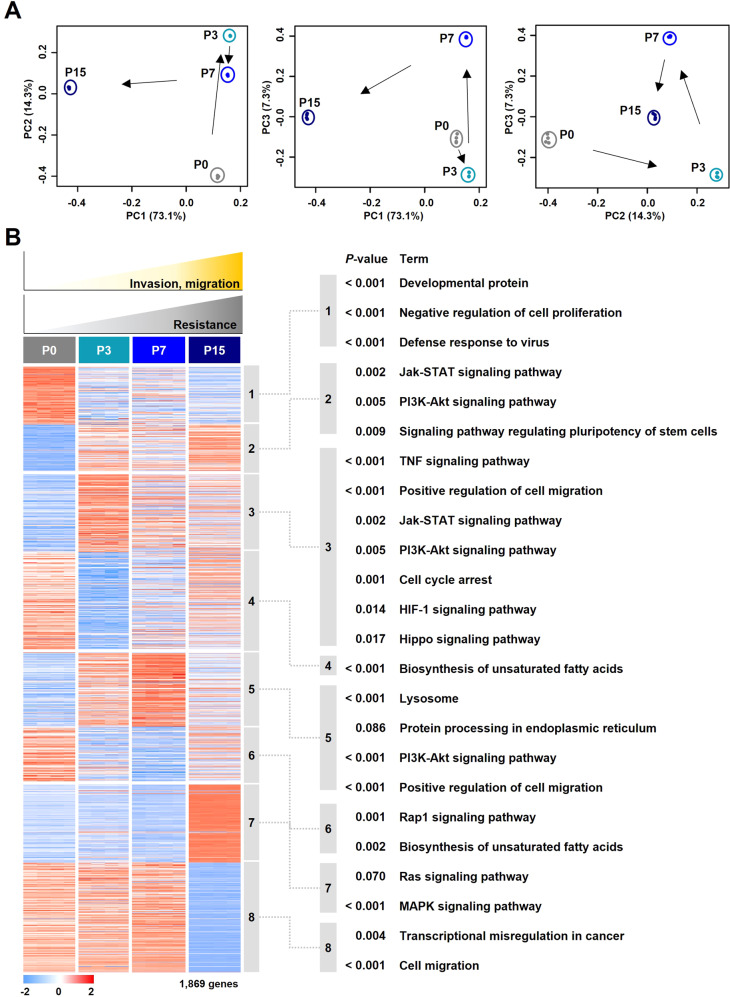
Overview of principal component analysis and differentially expressed gene analysis with the molecular mechanisms at four time-points. **A** The principal component analysis (PCA) of the GRC1 cell line (*n* = 16) is based on the 1869 genes associated with molecular mechanisms (CPM > 1 and S.D > 1). **B** Gene expression patterns of 1869 genes and enriched biological characteristics at four time-points. The colors in the heatmap reflect relatively high (red) and low (blue) expression (left panel). Results of Kyoto Encyclopedia of Genes and Genomes (KEGG) pathways and Gene Ontology (GO) enrichment analysis of four time-points (right panel).

**Fig. 4:**
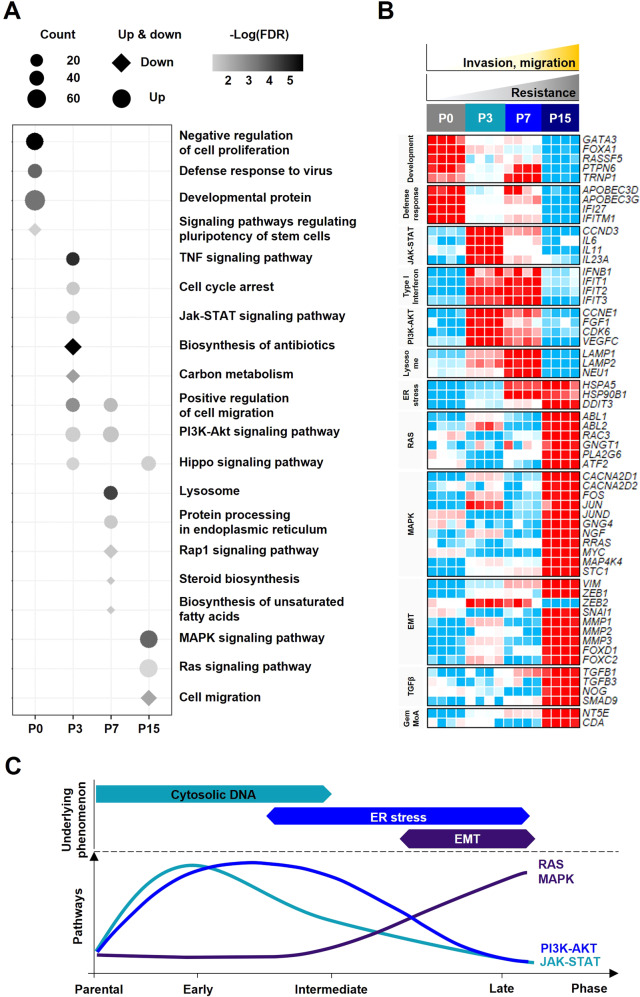
Significant biological characteristics and gene expression patterns representing four time-points. **A** Biological characteristics at each time-point. The size of the symbol indicates the number of genes on each term. The diamond and circle shapes indicated downregulation and upregulation, respectively. The darkness indicates the degree of FDR. **B** Gene expression patterns associated with significantly identified biological characteristics at each time-point. The colors in the heatmap reflect relatively high (red) and low (blue) expression CPM values. **C** Schematic graph of molecular changes in gemcitabine resistance. The top indicates the underlying phenomenon related to gemcitabine resistance. The bottom panel shows a graph of the signaling pathways that induce gemcitabine resistance. FDR false discovery rate, ER endoplasmic reticulum, EMT epithelial to mesenchymal transition, MoA mechanism of action.

Gene expression profile data of the GRC1 cell line were generated by RNA-sequencing technology to analyze the changes at four time-points. Differentially expressed 1,869 genes were selected with count per million and standard deviation (CPM > 1 and S.D > 1). Distribution in the two-dimensional space projected by principal component analysis (PCA) in the GRC1 cell line demonstrated a clear separation among all time-points. In particular, there was a difference of 73.1% in the distribution between parental phase P0 and late phase P15, and a significant difference was confirmed between early phase P3 and intermediate phase P7 (Fig. 3A). To investigate the biological characteristics of the GRC1 cell line, hierarchical clustering and functional analysis such as Gene Ontology (GO) and Kyoto encyclopedia of genes and genomes (KEGG) pathways were performed using each time-point related gene (Fig. 3B, Table S1). Based on the functional analysis, significant biological characteristics and genes representing the GRC1 cell line were selected (Fig. 4A, B). In parental phase P0, the development and negative regulation of cell proliferation related genes (*GATA3*, *FOXA1*, *RASSF5*, *PTPN6*, and *TRNP1*) and defense responses related genes (*APOBEC3D*, *APOBEC3G*, *IFI27*, and *IFITM1*) were upregulated. In early phase P3, JAK-STAT pathway (*CCND3*, *IL6*, *IL11*, and *IL23A*), type I IFN pathway (*IFNB1*, *IFIT1*, *IFIT2*, and *IFIT3*), and PI3K-AKT pathway (*CCNE1, FGF1, CDK6*, and *VEGFC*) were upregulated (Fig. S3A, B). In intermediate phase P7, the GRC1 cell line showed upregulation of genes related endoplasmic reticulum (ER) stress such as lysosomes (*LAMP1*, *LAMP2*, and *NEU1*) and unfolded protein response (*HSPA5*, *HSP90B1*, and *DDIT3*) (Fig. S3C). In late phase P15, RAS signaling pathway (*ABL1*, *ABL2*, *RAC3*, *GNGT1*, *PLA2G6*, and *ATF2*), MAPK signaling pathway (*CACNA2D1*, *CACNA2D2*, *FOS*, *JUN*, *JUND*, *GNG4*, *NGF*, *RRAS*, *MYC*, *MAP4K4*, and *STC1*), EMT-related genes (*VIM, ZEB1, ZEB2, SNAI1, MMP1, MMP2, MMP3*, *FOXD1*, and *FOXC2*), and TGF-β pathway (*TGFB1, TGFB3, NOG*, and *SMAD9*) were upregulated (Fig. S3D). The mRNA expression levels of early phase P3, intermediate phase P7, and late phase P15 related genes were confirmed by qRT-PCR (Fig. S3E). Additionally, *NT5E* and *CDA*, which are directly related to the gemcitabine mechanism of action, were increased continuously from parental phase P0 to late phase P15 (Fig. 4B). Various biological pathways were observed at each time-point, suggesting that the molecular mechanisms were induced in the GRC1 cell line to avoid gemcitabine treatment (Fig. 4C).

### Identification of key genes to develop a chemoresistance score

**Fig. 5:**
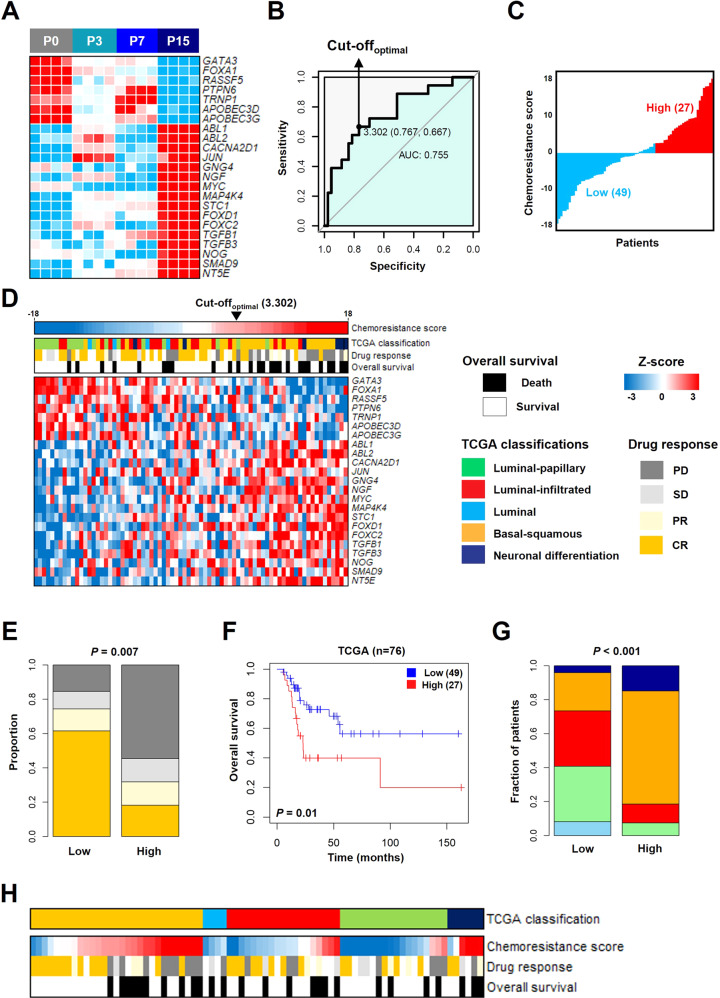
Identification of a chemoresistance score based on the GRC1 cell line and validation in TCGA. **A** Heatmap of the 23-gene signatures associated with gemcitabine resistance in the GRC1 cell line. The colors in the heatmap reflect relatively high (red) and low (blue) expression CPM values. **B** ROC curve of the sensitivity and specificity of 23-gene signatures for predicting complete response to gemcitabine treatment in the TCGA. **C** A chemoresistance score was calculated as the sum of each gene’s score, which was derived by multiplying the expression level of a gene by its corresponding coefficient. **D** Heatmap and clinical information of TCGA cohort (*n* = 76) grouped according to a chemoresistance score. The colors in the heatmap reflect relatively high (red) and low (blue). **E** The objective response rate to gemcitabine treatment was stratified into the two groups (*P* = 0.007 by the Fisher’s exact test). **F** Kaplan–Meier curve of two groups in TCGA cohort stratified by a chemoresistance score (*P* = 0.01 by the log-rank test). **G** Distribution of TCGA subtype stratified by chemoresistance score (*P* < 0.001 by the Fisher’s exact test). **H** A chemoresistance score, drug response, and overall survival according to TCGA subtype. ROC receiver operating characteristic, AUC area under the ROC curve, PD progressive disease, SD stable disease, PR partial response, CR complete response.

Among the activated biological pathways (Fig. 4A), 23 genes (*GATA3, FOXA1, RASSF5, PTPN6, TRNP1, APOBEC3D, APOBEC3G, ABL1, ABL2, CACNA2D1, JUN, GNG4, NGF, MYC, MAP4K4, STC1, FOXD1, FOXC2, TGFB1, TGFB3, NOG, SMAD9*, and *NT5E*) associated with the acquisition of gemcitabine resistance were selected and confirmed that the expression levels of genes differed compared to other phases (Fig. 5A). To apply the characteristics of GRC cell lines to clinical cohorts, a chemoresistance score was created from the sum of 23-gene’s values, which was derived by multiplying the expression level of a gene by its corresponding coefficient (Table S2). Receiver Operating Characteristic (ROC) analysis was performed to compare the sensitivity and specificity of complete response prediction of the 23-gene signatures for The Cancer Genome Atlas (TCGA) cohort with gemcitabine treatment (*n* = 76). Patients of TCGA cohort were classified by the area under the ROC curve (AUC = 0.755) and an optimal cutoff value (Cutoff optimal = 3.302, Fig. 5B). Based on a chemoresistance score, low and high score patients were represented by two groups (Fig. 5C, D). In addition, high score patients had significantly low response rates (*P* = 0.007 by the Fisher’s exact test, Fig. 5E) and poor prognosis (*P* = 0.01 by the log-rank test, Fig. 5F). To investigate the association between the TCGA molecular subtype and a chemoresistance score, the distribution of five molecular subtypes in each cluster was examined (Fig. 5G). In the basal-squamous subtype of the TCGA cohort, high score patients had lower objective response rates (Fig. 5H).

In addition, we found that the GRC1 cell line showed resistance to cisplatin and doxorubicin through in vitro experiments (Fig. S4A). Patients treated with other drugs (cisplatin, doxorubicin, and carboplatin) were analyzed according to the 23-gene signatures to determine the association between a chemoresistance score and response rate (Fig. S4B). The performance of a chemoresistance score was estimated (Fig. S4C). In the case of cisplatin treatment, high chemoresistance score patients had a significantly low response rate and poor prognosis (*P* = 0.004 by the Fisher’s exact test, *P* = 0.01 by log-rank test, respectively, Fig. S4D, E). Although statistical significance was insufficient due to the small number of events, a similar tendency was shown for carboplatin and doxorubicin treatments (Fig. S4D, E). Patients with NMIBC of UROMOL cohort [22] were analyzed according to the 23-gene signatures to determine the association between a chemoresistance score and disease progression (Fig. S5A, B, and C). High chemoresistance score patients had a poor prognosis (*P* < 0.001 by log-rank test, Fig. S5D). These results indicated that a chemoresistance score exhibited significant prognostic potential and predictive value for various chemotherapy in BC patients.

### Characteristics of gemcitabine-resistant-bladder cancer cell line in vivo

**Fig. 6:**
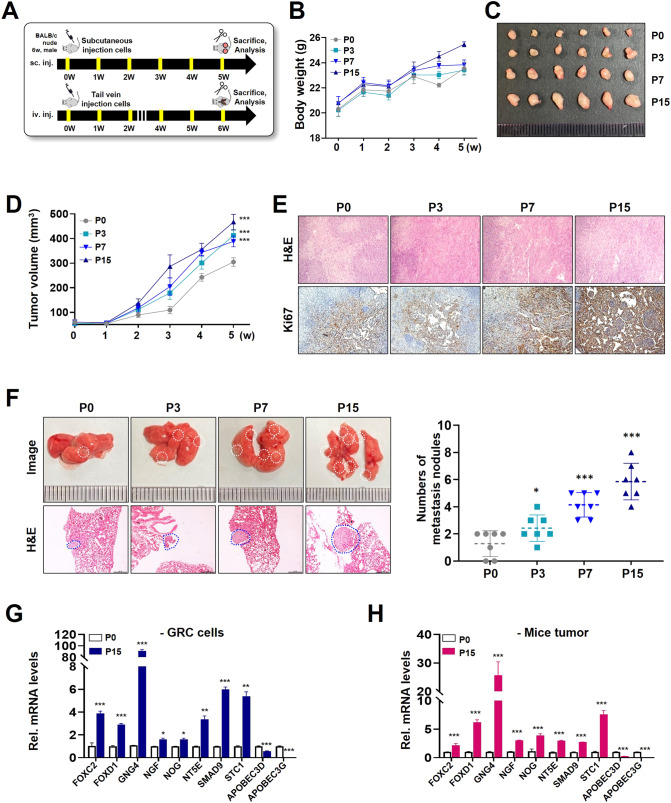
Effect of the GRC1 cell line on tumor proliferation and metastasis in mice. **A** Schematic description of the experimental schedule. **B** The graph shows the mouse body weight gains after the GRC1 cell line injection. **C** Representative images of tumors. Tumor tissues were surgically removed from nude mice 5 weeks post-injection. **D** Xenograft tumor volume of mice injected with parental phase P0 and the GRC1 cell line. **E** Representative images of H&E and Ki-67 expression by immunohistochemistry. **F** Representative lung images and H&E staining were obtained after injection of the GRC1 cell line via the tail vein (left). Comparison of the number of lung metastatic nodules formed after tail vein injection of the GRC1 cell line (right). Expression and validation of 10 genes out of 23-gene signatures **G** in the GRC1 cell line and **H** in tumor xenografts by qRT-PCR. *P < 0.05; **P < 0.01; ***P < 0.001.

According to the schematic diagram (Fig. 6A), the in vivo cancer characteristics of the GRC1 cell line on tumor growth and metastasis were confirmed (Fig. 6B–F). For tumor growth analysis, the GRC1 cell line was subcutaneously injected into the mouse flank or tail vein for lung metastasis analysis using BALB/c nude mice, and the mice were sacrificed 5–6 weeks later. As a result, the body weight of the mice injected with the GRC1 cell line was slightly increased compared to that of the mice injected with parental phase P0 (Fig. 6B). The size and volume of the tumor were significantly increased in mice injected with the GRC1 cell line (Fig. 6C, D). Consistent with these results, the level of Ki67 staining, a marker of cell proliferation, was higher in the tumor tissue sections obtained from the xenografts of the GRC1 cell line than in parental phase P0 (Fig. 6E). The injection of the GRC1 cell line representing lung metastasis, produced more lung nodules, of which late phase P15 mouse group increased by approximately 6 times compared to parental phase P0 mouse group (Fig. 6F). Comparing parental phase P0 and late phase P15, 10 genes out of 23-gene signatures were randomly selected and confirmed in the cancer tissue and mouse xenografts obtained after sacrificing the mice. The expression levels of *FOXC2, FOXD1, GNG4, NGF, NOG, NT5E, SMAD9*, and *STC1* were significantly higher in late phase P15 cells than in parental phase P0. On the other hand, the expression levels of *APOBEC3D* and *APOBEC3G* were significantly lower (Fig. 6G). The same results were confirmed in the tumor tissues of the mouse model (Fig. 6H). Taken together, we confirmed that the GRC1 cell line has proliferative, invasive, and migratory abilities even in vivo experiments using mice. Therefore, it will be possible to identify chemotherapy resistance using the gene signature of the GRC1 cell line model.

## Discussion

Although considerable efforts have been made in previous studies to understand the associations between chemotherapy resistance and malignancy of BC, the mechanisms involved are not fully understood, and chemoresistance remains a challenge in BC patients [23, 24]. To address this problem, an integrated study has been reported on the inherent biological mechanisms of human diseases, including cancer, or stepwise changes in chemoresistance acquisition over time [25–29]. In the case of Gemcitabine, which is widely used as an anticancer drug, many bladder cancer patients did not benefit from chemotherapy due to resistance after continuous treatment. In previous studies, it was reported that cancer cells that acquired chemotherapy resistance increased cell growth compared to previous cells, and higher expression of genes involved in DNA damage response (DDR) was observed [12]. In addition, it has been reported that metastasis and chemotherapy resistance are closely related [12, 28]. Although many studies compare the molecular mechanisms at two time-points before and after the acquisition of anticancer drug resistance, it will be more important to clarify the sequential molecular mechanism of chemoresistance and to identify biomarkers for better treatment of BC [9, 16, 17]. Therefore, we established sequential GRC cell lines to understand acquiring chemotherapy resistance (Fig. 1 and Fig. S2). Similarly, the GRC cell lines showed increased cell viability and colony numbers in vitro (Fig. S2). The GRC cell lines showed a sequentially increased cell motility in a 3D environment as well as in vitro and the expression levels of EMT-related mRNA and protein were also increased (Fig. 2).

Gene expression profile data (RNA-seq) on the GRC1 cell line was generated at four time-points. The PCA and time-point analysis of the GRC1 cell line indicated the possibility of molecular changes for cells to evade chemotherapy treatment (Fig. 3). In early phase P3, upregulation of type I IFN pathway, IL-6 family, JAK-STAT pathway, and PI3K-AKT pathway seems to be related to cytosolic DNA reactions. The activation of the JAK-STAT pathway and PI3K-AKT pathway could induce cell survival and chemoresistance (Fig. 4) [30–33]. In intermediate phase P7, DNA damaged by drug treatment might synthesize abnormal proteins and provoke ER stress. This event allows the subsequent induction of unfolded protein response (UPR), which could restore ER homeostasis and contribute to chemotherapy resistance [34]. Activation of type I IFN and UPR could enhance the PI3K-AKT pathway and consequently induce the lysosomal pathway [35, 36]. Lysosomes can be closely related to extracellular matrix interactions and increases in cell migration by lysosomal exocytosis. In particular, *LAMP1* and *LAMP2*, which were major components of the docking of lysosomal exocytosis, were highly upregulated in intermediate phase P7 (Fig. 4) [37]. Accumulation of DNA damage and ER stress leads to activation of biological pathways (EMT, TGF-β pathway, RAS pathway, and MAPK pathway) in late phase P15, and these pathways are commonly associated with mesenchymal stem cells, cancer progression, metastasis, and chemotherapy resistance [38]. Additionally, upregulation of *NT5E* and *CDA* plays an important role in resistance acquisition in the gemcitabine action mechanism (Fig. 4) [9].

To develop a chemoresistance score, we establish the 23-gene signatures which are associated with chemoresistance in many cancers. *GATA3*, *FOXA1*, *RASSF5*, *PTPN6*, and *TRNP1*, involved in the luminal markers and negative regulation of cell proliferation, are related to tumor suppression and chemotherapy resistance [39–41]. *APOBEC3D* and *APOBEC3G*, involved in the antiviral response pathway, are related to chemoresistance and metastasis [42]. *ABL1* and *ABL2*, components of the RAS pathway, are associated with chemotherapy resistance [43]. The overexpression of *CACNA2D1*, *JUN*, *GNG4, NGF, MYC*, and *MAPK4K4*, components of the MAPK pathway, is associated with tumor aggressiveness and chemotherapy resistance [44–47]. *STC1* is related to chemoresistance, invasion, and metastasis in breast cancer [48]. *FOXD1* and *FOXC2*, composed of the FOX family of transcription factors, have critical roles in the regulation of cell development and chemoresistance [49, 50]. *TGFB1*, *TGFB3*, *NOG*, and *SMAD9*, components of the TGF-β pathway, participate in tumor metastasis and cancer stem cells [51, 52]. *NT5E* is one of the gemcitabine action mechanisms that is associated with chemoresistance and poor prognosis in breast cancer [53] (Fig. 5A). Based on a chemoresistance score, low and high score patients were represented by two groups (Fig. 5D). In addition, high score patients had significantly low response rates and poor prognosis (Fig. 5E, F). Since chemotherapy is known to be effective in patients with the basal-squamous subtype of TCGA cohort [54], we have validated a potential therapeutic benefit in the subtype and progression of NMIBC patients according to a chemoresistance score (Fig. 5H and Fig. S5). In addition, the tumor growth and metastatic ability of the GRC1 cell line were confirmed in vivo, and the expression levels of 10 selected genes were confirmed in cancer tissues generated by mouse xenografts (Fig. 6). Consequently, our gene signatures could be used to predict a patient’s response to gemcitabine.

Taken together, this process is not independent but is dependent on cancer progression mechanisms related to anticancer drug resistance. These results are a strategy for bladder cancer cell lines against stress (or driving force) that are built up in cells over time. In this study, we established the sequential molecular mechanism changes of GRC cell lines of BC and analyzed gene signatures at each phase based on multiple markers. We suggest that our findings improve prognosis by predicting to acquiring of chemotherapy resistance in BC patients.

## Materials and methods

### Cell lines

The human bladder cancer cell line, T24 was purchased from American Type Culture Collection (ATCC, VA, USA). All cell lines, including T24 parental cells and stepwise gemcitabine-resistance cancer cell lines, were cultured in Dulbecco’s modified Eagle’s medium (DMEM) with 10% FBS and 1% penicillin/streptomycin (Capricorn Scientific GmbH, Germany). All cell lines were incubated at 37 °C in a humidified atmosphere of 5% CO_2_ and tested for mycoplasma on a bimonthly schedule.

### Construction of sequential gemcitabine-resistance cancer cell lines

To establish sequential gemcitabine-resistance cancer (GRC) cell lines, parental T24 cells were treated with 1.5 μM gemcitabine (Eli Lilly and company, IN, USA) in a culture medium for 72 h when cells reached a confluence of ~90% (Fig. 1A). DMEM containing gemcitabine was replaced, and viability was monitored every 2–3 days. When the GRC cell lines proliferated to form colonies, they further proliferated in DMEM and were named Phase 1 (P1). The constructed P1 was further cultured to make cell stocks, and some cells were moved to a medium containing gemcitabine to build the next phase. In the same methods, the GRC cell lines from parental phase P0 to late phase P15 were constructed. A total of four types of GRC cell lines were constructed and named GRC1-4.

### Cell survival assay

Parental phase P0 and the GRC cell lines were seeded into 96-well plates with 1 × 10^3^ cells per well. After 24 h, the cells were exposed to gemcitabine, for 0, 24, 48, and 72 h. Then, 3-(4,5-dimethylthiazol-2-yl)-2,5-diphenyltetrazolium bromide (Sigma–Aldrich, MO, USA) was added to each well followed by incubation. After 1 h, DMEM was added to 100 μl dimethyl sulfoxide (DMSO; Duchefa Biochemie, Netherlands) in each well to dissolve the formazan crystals. The absorbance value at 540 nm was examined by a spectrophotometer microplate reader (Victor3), and cell viability was calculated as a percent compared to control cells. The GRC cell lines (1 × 10^3^ cells/well) were seeded on a 96-well plate and cultured until ~60–70% confluent, and then drug treatment was performed. Dose-response curves of various concentrations of gemcitabine, cisplatin, and doxorubicin [μM] were depicted, and the IC_50_ was calculated using GraphPad Prism 9 (GraphPad Software, USA) with non-linear (curve fit) regression algorithms.

### Anchorage-dependent and independent-growth assays

For the anchorage-dependent growth assay, the GRC cell lines were seeded in 6-well plates at a density of 1 × 10^3^ cells per well and incubated for ~7 days until visible colonies were formed. Colonies were stained with 4% formaldehyde and 0.1% crystal violet solution for 1 h, respectively. The number of colonies was counted manually using ImageJ (NIH; National Institutes of Health, USA). Anchorage-independent growth of cells was examined by the survival of colonies on soft agar as described previously [55]. The plates were incubated for 10 days, and colonies were scored by microscopy.

### Cell invasion and migration assay

Cell invasion and migration ability were measured using a Boyden chamber, as previously described [55]. Briefly, 4 × 10^4^ cells in serum-free medium were loaded on matrigel (BD Biosciences, CA, USA) or collagen-coated membranes, and incubated for 24 h. DMEM containing 1% FBS was added to the lower chamber.

### Wound-healing assay

The GRC cell lines were seeded on six-well plates and cultured for 24 h until 90% confluence. After making wounds on the surface of the board with the yellow tip of a P200 pipette, the cells were washed several times with phosphate-buffered saline (PBS) to remove cell debris. Then, the cells were cultured in 5% CO_2_ at 37 °C. After 20 h, the cells were visualized by light microscopy. After, photographs of the scratched area were taken at intervals. Three random fields were marked and measured. The migration index was expressed as the ratio of the migrating distance of treated cells to that of control cells.

### Immunostaining and migration analysis of bladder cancer cells cultured in 3D conditions via microfluidic devices

The design and fabrication method of the microfluidic device and a recipe for 3D cell culture were described in previous reports [18, 19, 56]. The central channel of the microfluidic device was filled with collagen type I solution (2 mg/ml), and the medium channels were coated with collagen solution (35 μg/ml in PBS) to enhance cell attachment on the device surface. After rinsing with fresh medium, a suspension of bladder cancer cells (1 × 10^6^ cells/ml) under each phase was introduced into the medium channels. Bladder cancer cells were cultured in the microfluidic device, and we assessed the migration of cells on Day 5 of culture. The cells were fixed with 4% paraformaldehyde and permeabilized with 0.1% Triton X-100. Actin filaments and nuclei were stained with phalloidin-594 (1:400, Invitrogen, CA, USA) and Hoechst 33342 (1:2000, Thermo Fisher Scientific, MA, USA), respectively. The entire device was scanned, and images were obtained using a high content screening microscope (CELENA X, Logos Biosystems, Korea). The 8–9 region of interest (ROI) images of three different devices were taken (total *n* = 25), and all binary and threshold images were analyzed quantitatively by using ImageJ software regarding the maximum filtration distance of cancer cells, the infiltration area, and the infiltrated cancer cell numbers. To assess the infiltrated areas, the proportion of the fluorescence pixels was calculated in ROI images.

### Quantitative real-time polymerase chain reaction

Total RNA was prepared using the RNeasy preparation kit (Qiagen, MD, USA) according to the manufacturer’s instructions. Total RNA (1000 ng) was analyzed using the PrimeScript^TM^ RT reagent Kit (Takara, Shiga, Japan) according to standard protocols. qRT-PCR was performed using TB Green Premix Ex Taq (Takara, Shiga, Japan) and a CFX96 real-time PCR detection system (BioRad, CA, USA). The reproducibility of the quantitative measurements was evaluated by three independent cDNA syntheses and PCR amplification from each preparation of RNA. For mRNA analysis, data were normalized to GAPDH as an endogenous loading control, and fold changes were calculated via relative quantification (2^−ΔΔCt^). A detailed list of qRT-PCR primer sequence information is provided in Table S3.

### Immunoblotting

Whole-cell protein extracts were lysed with radioimmunoprecipitation (RIPA) buffer supplemented with protease inhibitors (Roche, IN, USA) and then centrifuged at 4 °C, 12,000 × *g* for 15 min. The protein concentration was determined with a BCA assay (Thermo Fisher Scientific, MA, USA). Then, extracted proteins were subjected to 10–12% SDS-polyacrylamide gel electrophoresis (SDS-PAGE) and electrically transferred onto a nitrocellulose (NC) membrane (GE Healthcare, Denmark). After incubation with the target primary antibody (GAPDH, cell signaling #2118; MMP-1, Santa Cruz #sc-137044; MMP-2, cell signaling #cs4022; MMP-9, cell signaling #cs3852; NCAD, cell signaling #cs4061; VIM, Santa Cruz #sc-6260; SNAIL, Santa Cruz #sc-271977; and ECAD, cell signaling #cs3195) at 4 °C overnight, the cells were treated with ECL reagent, and exposed to X-ray film to visualize the bands of interest.

### RNA extraction and RNA-seq data processing

Total RNA extraction was performed using the RNeasy Mini Kit (Qiagen, MD, USA) according to the manufacturer’s instructions. Comparability of quantities and RNA quality were measured by NanoDrop 2000 (Thermo Fisher Scientific, MA, USA) and confirmed by electrophoresis on a 6% formaldehyde gel, followed by ethidium bromide staining (EtBr). Library construction for whole transcriptome sequencing was performed using a kit. Sequencing was performed in paired-end reads (2 ×200 bp) using a HiSeq2500 (Illumina, CA, USA). Reference genome sequence data from Homo sapiens were obtained from the Ensemble genome browser (assembly ID: GRCh38). The reference genome indexing and read mapping of samples were performed using STAR software (ver. 2.6.1a) [57]. FeatureCounts (ver. 1.6.2) software was used to calculate the generated binary alignment map files [58]. The RNA-sequencing data were calculated to CPM values, normalized using quantile normalization, log2-transformed, and median centered across genes and samples. The dataset is available in the National Center for Biotechnology Information (NCBI) Gene Expression Omnibus (GEO) public database under data series accession number GSE190636.

### Gene expression profile data and chemoresistance score

Differentially expressed genes were selected with expression levels and standard deviation (CPM > 1 and S.D > 1) and used principal component analysis (PCA) to compare the differences in the GRC1 cell line. To explore highly enriched functions, we performed GO and KEGG pathway analysis using the Database for Annotation, Visualization and Integrated Discovery (DAVID) tool (http://david.ncifcrf.gov) with significance criteria (*P* < 0.001 and false discovery rate < 0.25). Pathview (ver. 1.34.0) tool was used to visually represent the analysis [59]. Gene expression profile data and the clinical information on bladder cancer patient cohort provided by TCGA (*n* = 407) consortium were downloaded from Cancer Browser (https://xena.ucsc.edu), and we used 185 samples with gemcitabine (*n* = 76), cisplatin (*n* = 75), carboplatin (*n* = 22), and doxorubicin (*n* = 12) records. To develop a chemoresistance score, we identified candidate genes between parental phase P0 and late phase P15 in the GRC1 cell line and adopted a developed strategy that uses coefficients of the Cox linear regression model. The gene-specific coefficient values were derived from the number of colonies of the GRC1 cell line at four time-points. A chemoresistance score was created from the sum of each gene’s score, which was derived by multiplying the expression level of a gene by its corresponding coefficient. Samples of the clinical cohort were classified based on the area under ROC curve (AUC) values derived from Receiver Operating Characteristic (ROC) analysis.

### Animal study

For the subcutaneous model, cell suspensions (2 × 10^6^ cells) mixed with Matrigel (Corning, NY, USA) were subcutaneously injected into the flank region of 4 weeks old BALB/c nude mice. Tumor size was measured every week from the time when external size measurement was possible, and the tumor volumes were calculated as follows: V (mm^3^) = length (mm) × width^2^ (mm^2^). For the lung metastasis model, cell suspensions (1 × 10^7^ cells) in PBS were injected through the tail vein. Mice were sacrificed 5 weeks later to confirm tumors. Some tumors were sectioned for immunohistochemistry and others were used for qRT-PCR. Lung tissues were fixed in formalin and sectioned for H&E staining, and immunohistochemistry. The mice were sacrificed and manipulated by the protocol approved by the Animal Care Committee of Kosin Medical University, Korea.

### Immunohistochemistry

Tissue microarray (TMA) blocks were selected tissue cores (diameter 2 mm) from mouse tumor paraffin blocks. To make slides for IHC, all tissue samples were fixed in buffered formalin (Sigma–Aldrich, MO, USA) and embedded in paraffin. Paraffin-embedded tissues were deparaffinized in xylene and rehydrated in graded alcohol (100, 90, 80, and 60%). Antigen retrieval (10 min in boiling water) was performed, and sodium citrate was used as the retrieval buffer. The primary Ki67 antibody used was rabbit monoclonal IgG (Abcam, MA, USA). Immunostaining was performed with a Rabbit IgG/Mouse IgG Vectastain Elite ABC Kit (Vector Laboratories, CA, USA). Vectastain Elite ABC Reagent was added (30 min at RT), and immunoreaction was detected using 3,3’-diaminobenzidine (Sigma–Aldrich, MO, USA) as a chromogen. Then, the TMA slides were counterstained with Mayer’s hematoxylin (Dako, CA, USA).

### Statistical analysis

All analyses were performed at least three times and represented as the mean ± standard deviation (S.D). Clustering analysis with the centered correlation coefficient and centroid linkage method was performed using the ComplexHeatmap (ver. 2.4.3) tool. To estimate the significance of differences between subgroups, two sample *t-*tests and Fisher’s exact test were performed. The Kaplan–Meier method was used to calculate overall survival was assessed using the log-rank test. All statistical analyses were performed by the R language environment (ver. 3.6.3).

## Supplementary information

original data files

Supplementary materials

## Data Availability

The dataset generated by RNA-seq is available in the Gene Expression Omnibus (GEO) database under data series accession number GSE190636.
