# Supplementary figures and images for: Stepwise molecular mechanisms responsible for chemoresistance in bladder cancer cells
Source: Cell Death Discov. 2022 Nov 7;8:450. doi: 10.1038/s41420-022-01242-8 (PMC9640638; doi:10.1038/s41420-022-01242-8)

# Supplementary figure S6

(Figure 2D uncropped full blot)

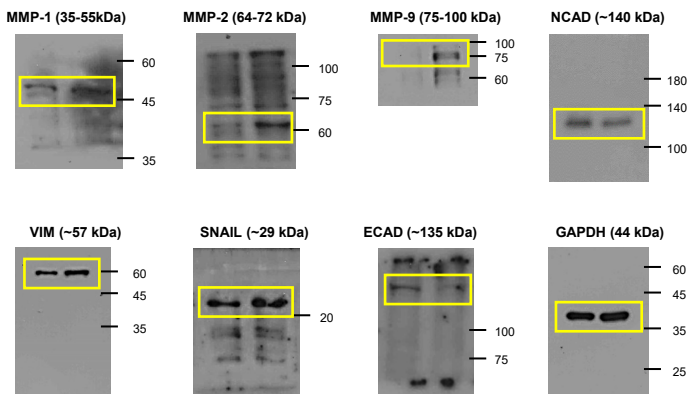

Supplement: Supplementary file 1 — original data files [file 41420_2022_1242_MOESM1_ESM.pdf]
