# Supplementary material for: Stepwise molecular mechanisms responsible for chemoresistance in bladder cancer cells
Source: Cell Death Discov. 2022 Nov 7;8:450. doi: 10.1038/s41420-022-01242-8 (PMC9640638; doi:10.1038/s41420-022-01242-8)
Supplement: Supplementary file 2 — Supplementary materials [file 41420_2022_1242_MOESM2_ESM.pdf]

Supplementary materials

Supplementary figure S1

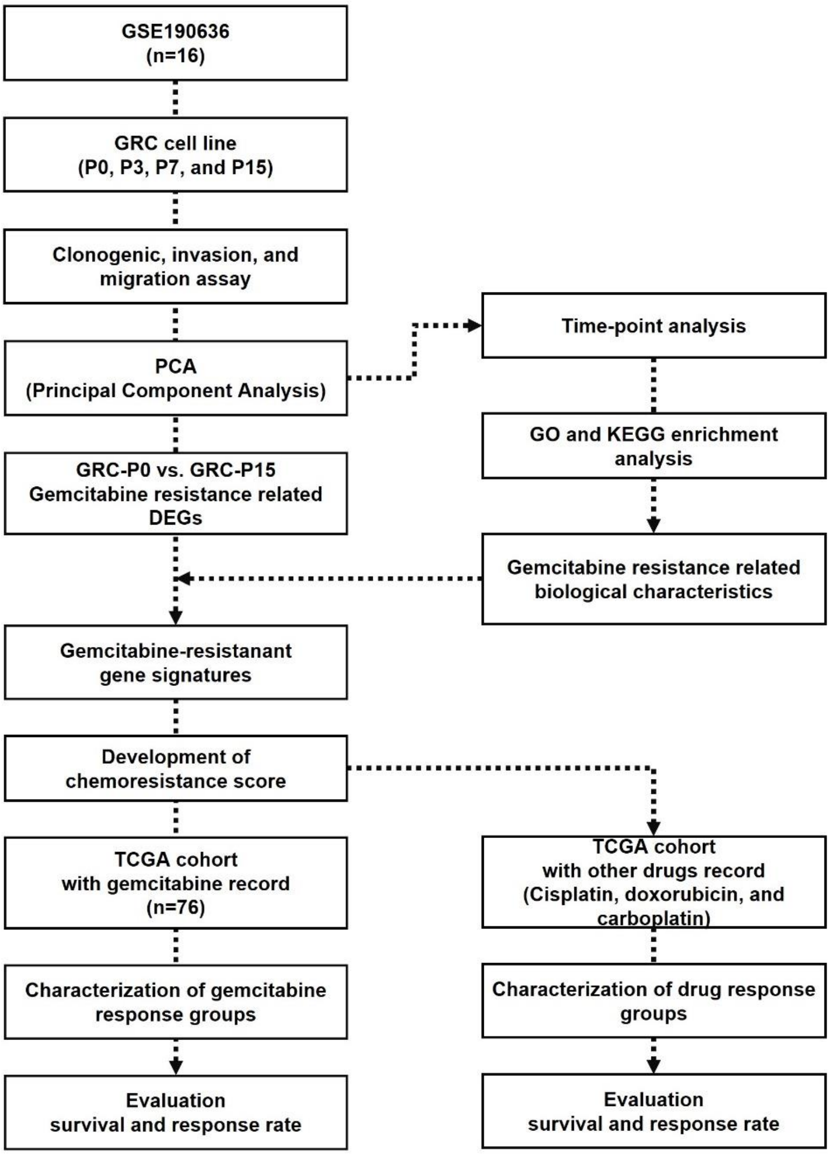

Supplementary Figure S1. Workflow diagram to characterize the molecular mechanisms of gemcitabine-resistant cancer (GRC) cell line. GRC, Gemcitabine-resistance cancer; DEGs, differentially expressed genes; GO, Gene Ontology; KEGG, Kyoto encyclopedia of genes and genomes; TCGA, The Cancer Genome Atlas.

Supplementary figure S2

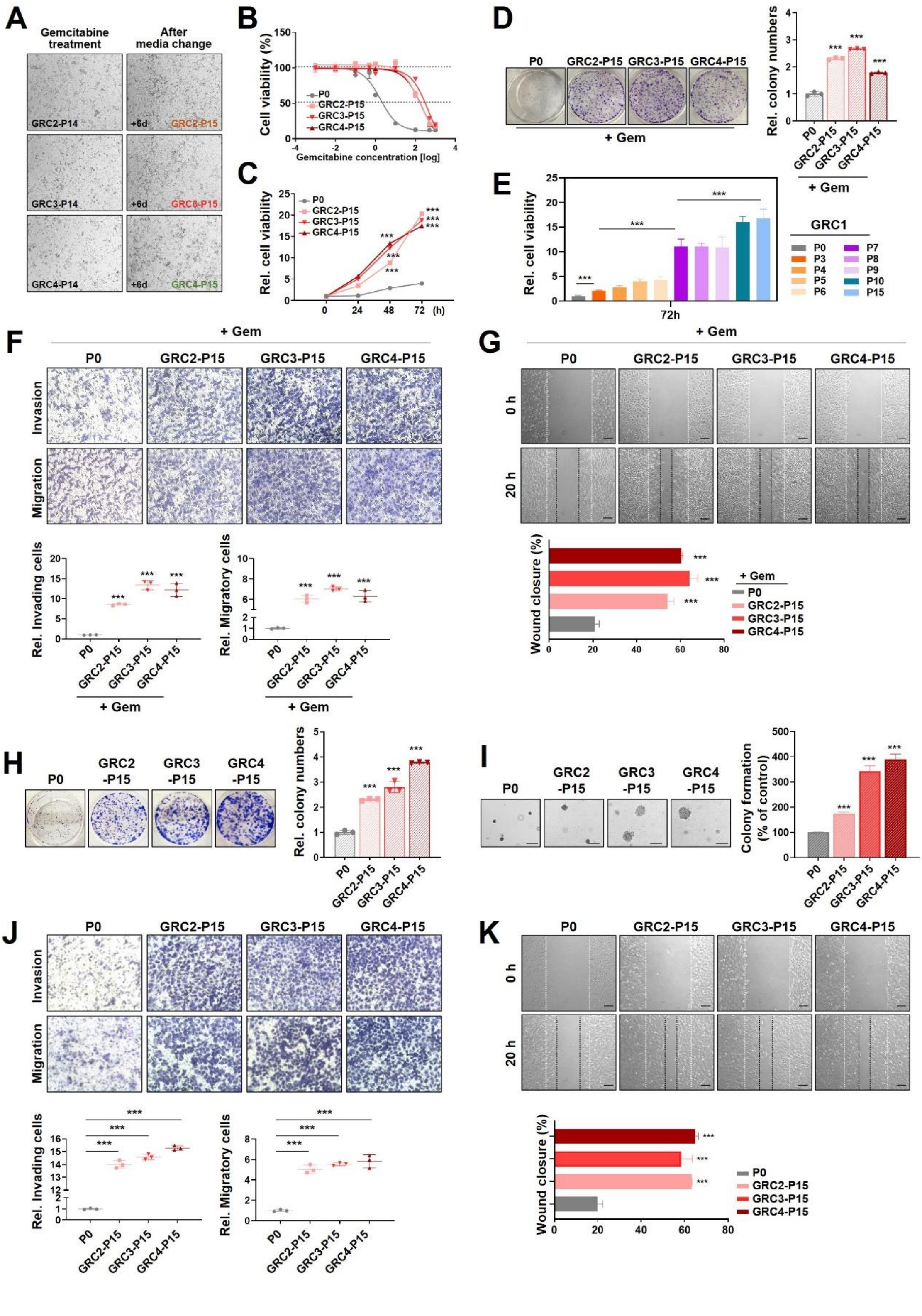

Supplementary Figure S2. Characterization of sequential GRC2-4 cell lines. A Representative picture of the establishment phases of gemcitabine treated P15 in P14 of the GRC2-4 cell lines is presented (d; day). **B** Gemcitabine sensitivity curves were obtained with MTT assays after 72h of treatment with various gemcitabine concentrations. **C** Cell survival assays were performed to determine the cell viability of P15 of the GRC2-4 cell lines compared to parental phase P0 at 0, 24, 48, and 72 h. **D** Anchorage-dependent growth assay was performed in the P15 of the GRC2-4 cell lines with treated 300 nM of gemcitabine. **E** Cell viability is presented at the 72h time-points from early phase P3 to late phase P15 of the GRC1 cell line compared to parental phase P0. **F** Cell invasion and migration abilities of late phase P15 of the GRC2-4 cell lines with 300 nM of gemcitabine were enhanced compared to parental phase P0. Cells were detected and calculated by counting cells per field. ×400. **G** A wound-healing assay was performed to examine the ability of wound closure of late phase P15 of the GRC2-4 cell lines with treated 300 nM of gemcitabine compared to P0. Monolayers were scratched using a 200 μl pipette tip and then photographed at 0 h and 20 h. The extent of wound healing was quantified using ImageJ software and the percentage of wound closure was calculated. **H-I** Anchorage-dependent (**H**) and anchorage-independent (**I**) growth assays were performed in P15 of the GRC2-4 cell lines, and P0 for 7 days. **J** Cell invasion and migration abilities of P15 of the GRC2-4 cell lines were enhanced compared to P0. Cells were detected and calculated by counting cells per field. ×400. **K** A wound-healing assay was performed to examine the ability of wound closure of P15 of the GRC2-4 cell lines compared to parental phase P0. ***, $P < 0.001$.

Supplementary figure S3

A

JAK-STAT pathway – P3

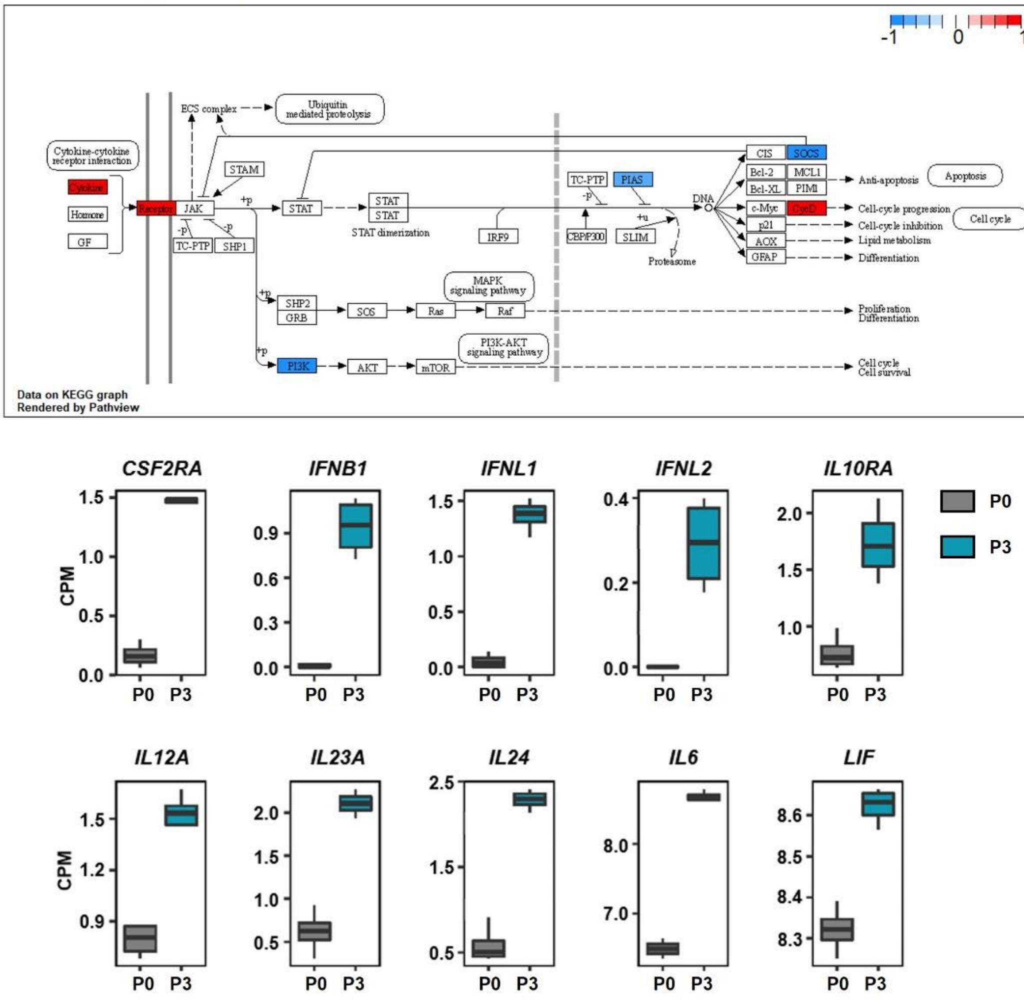

B

PI3K-AKT pathway – P3/P7

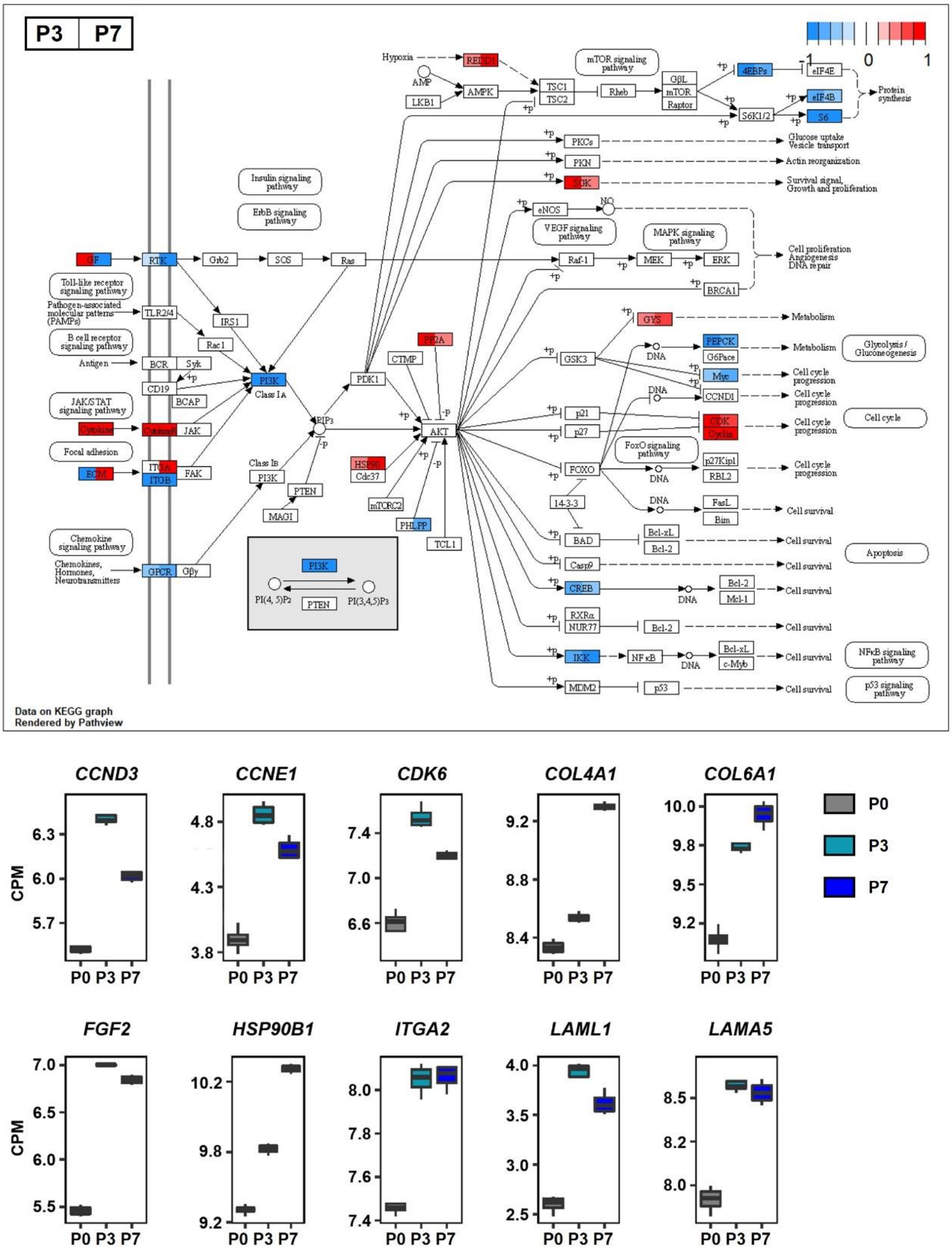

C

Lysosome – P7

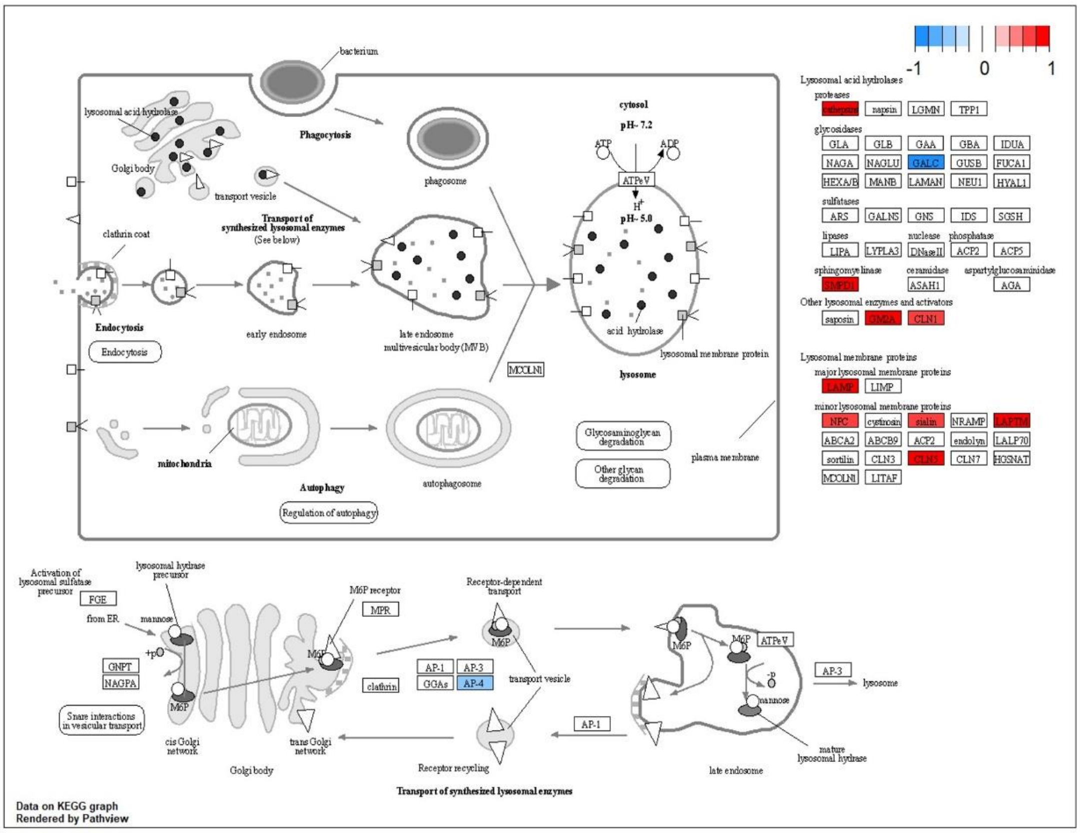

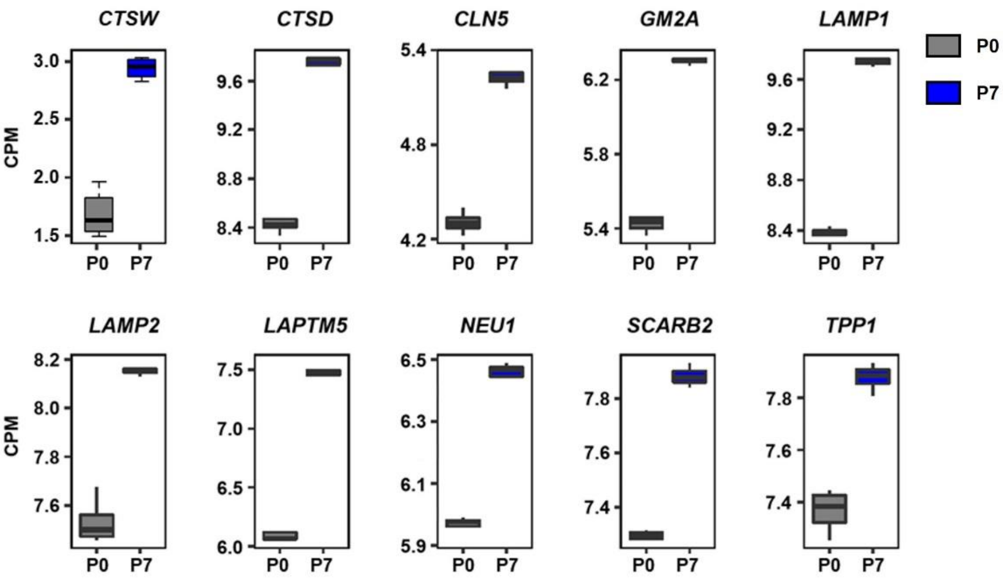

D

MAPK pathway – P15

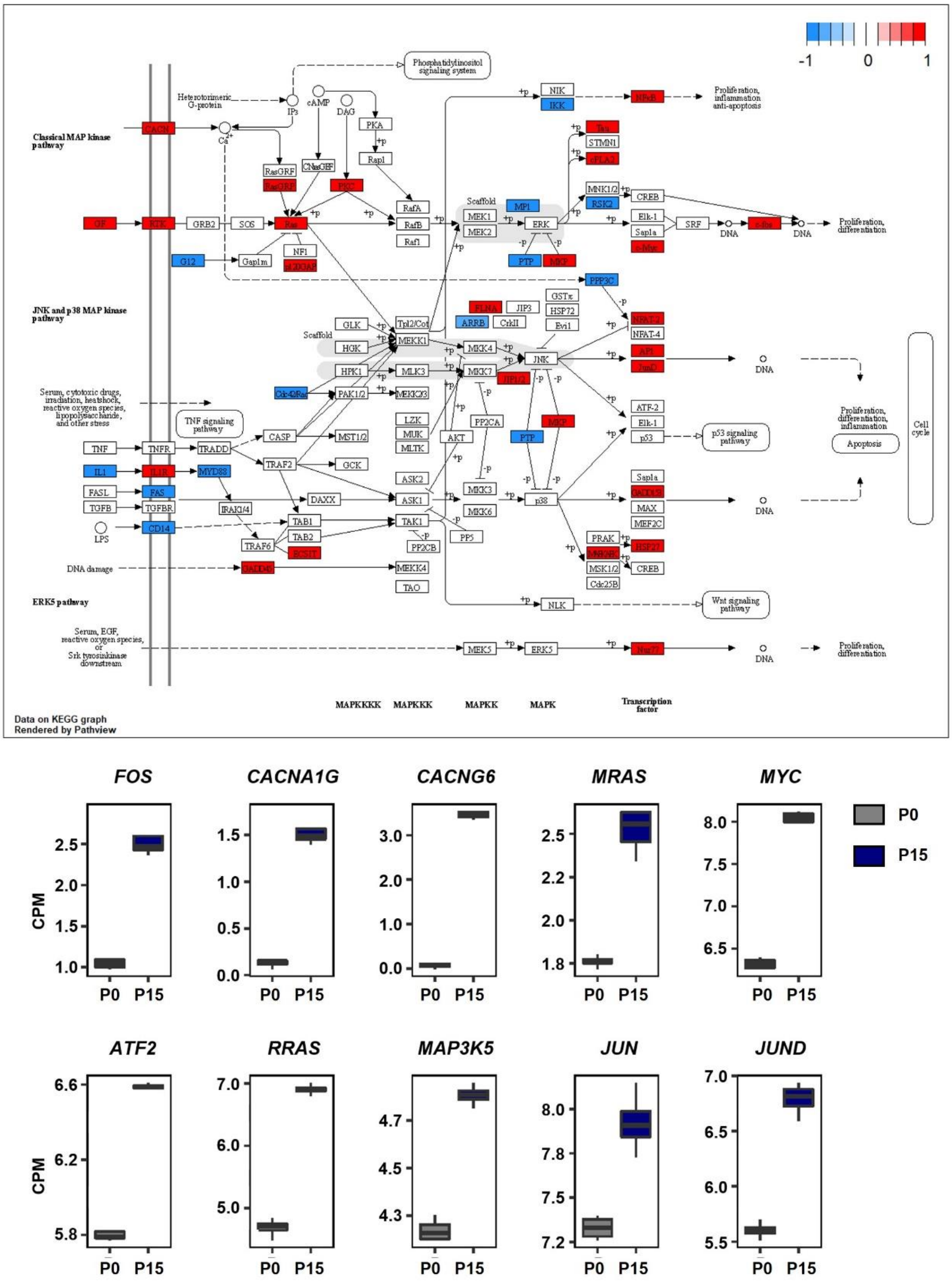

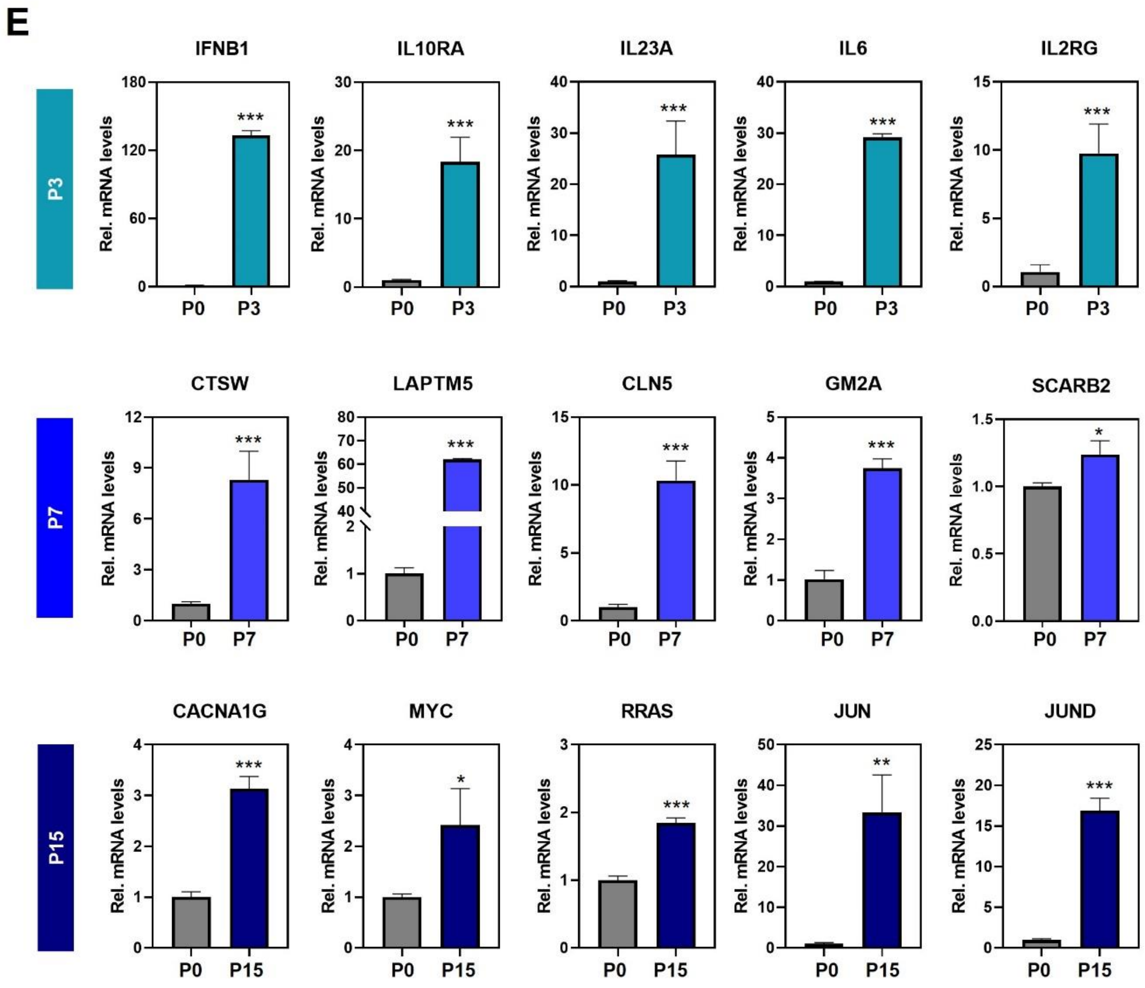
E
P3
IFNB1
IL10RA
IL23A
IL6
IL2RG
P7
CTSW
LAPTM5
CLN5
GM2A
SCARB2
P15
CACNA1G
MYC
RRAS
JUN
JUND
Rel. mRNA levels
P0
P3
P7
P15
***
**
*

Supplementary Figure S3. Pathview analysis and boxplots of the gene expression level. A JAK-STAT signaling pathway in early phase P3 and boxplots of the related genes expression level between parental phase P0 and early phase P3. **B** PI3K-AKT signaling pathway in early phase P3 and intermediate phase P7 and boxplots of the related genes expression level between in parental phase P0, early phase P3, and intermediate phase P7. **C** Lysosomes in intermediate phase P7 and boxplots of the related genes expression level between parental phase P0 and late phase P15. **D** MAPK signaling pathway in late phase P15 and boxplots of the related genes expression level. The colors in the box reflect activation (red) and inactivation (blue). **E** The mRNA expression levels of early phase P3, intermediate phase P7, and late phase P15 related genes were confirmed by qRT-PCR. *, $P < 0.05$; **, $P < 0.01$; ***, $P < 0.001$.

Supplementary figure S4

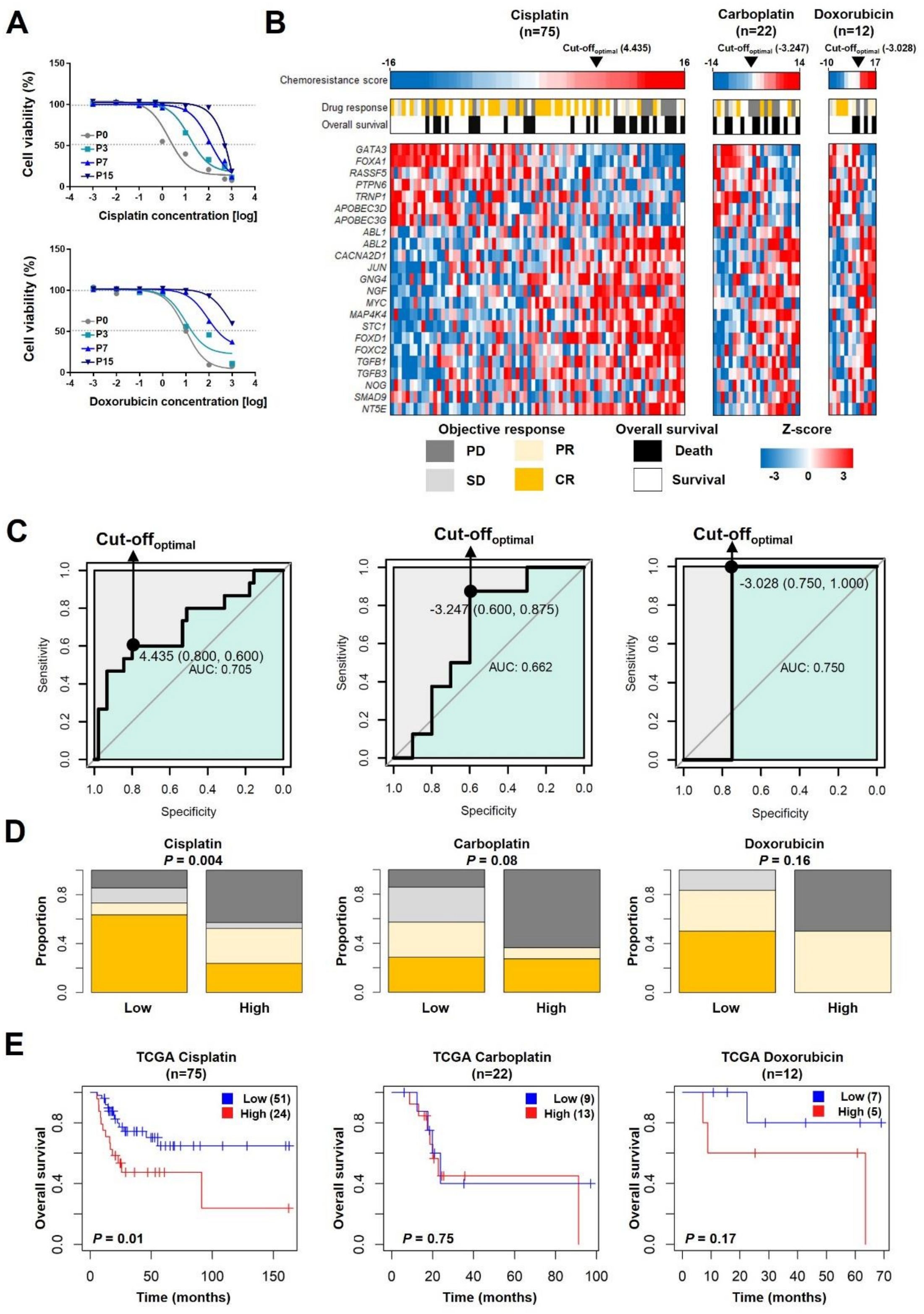

Supplementary Figure S4. Chemoresistance score to other drugs. A Drug sensitivity curves were obtained with MTT assays after 72 h of treatment with cisplatin and doxorubicin [μM]. **B** Heatmap, drug response, and overall survival of TCGA cohort grouped according to a chemoresistance score. The colors in the heatmap reflect relatively high (red) and low (blue). **C** ROC curve of the sensitivity and specificity of a chemoresistance score for predicting complete response to cisplatin, carboplatin, and doxorubicin. **D** The objective response rate to other drugs was stratified into the two groups ($P = 0.004$, $P = 0.08$, and $P = 0.16$ by the Fisher's exact test, respectively). **E** Kaplan-Meier curve of two groups in TCGA cohort stratified by a chemoresistance score ($P = 0.01$, $P = 0.75$, and $P = 0.17$ by the log-rank test, respectively). SEM, standard error of the mean; ROC, Receiver Operating Characteristic; AUC, area under the ROC curve; PD, progressive disease; SD, stable disease; PR, partial response; CR, complete response.

Supplementary figure S5

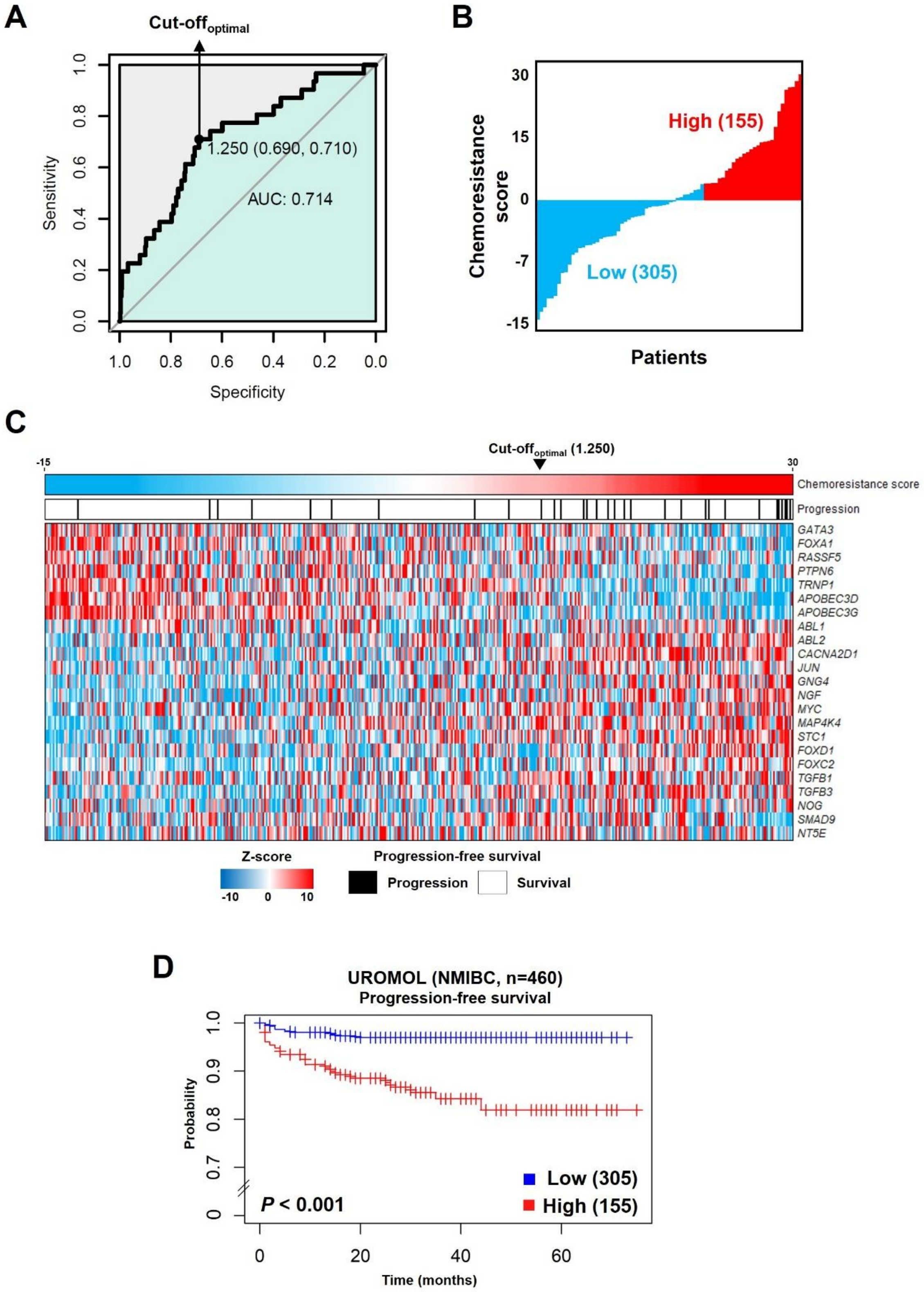

Supplementary Figure S5. Gene expression pattern and survival analysis for NMBIC patients in UROMOL cohort based on a chemoresistance score. A ROC curve of the sensitivity and specificity of 23-gene signatures for predicting progression of NMBIC patients in the UROMOL cohort. **B** A chemoresistance score was calculated as the sum of each gene's score, which was derived by multiplying the expression level of a gene by its corresponding coefficient. **C** Heatmap of the UROMOL cohort (n=460) grouped according to a chemoresistance score. The colors in the heatmap reflect relatively high (red) and low (blue). **D** Kaplan-Meier curve of two groups in the UROMOL cohort stratified by a chemoresistance score ($P < 0.001$ by the log-rank test).

Supplementary figure S6

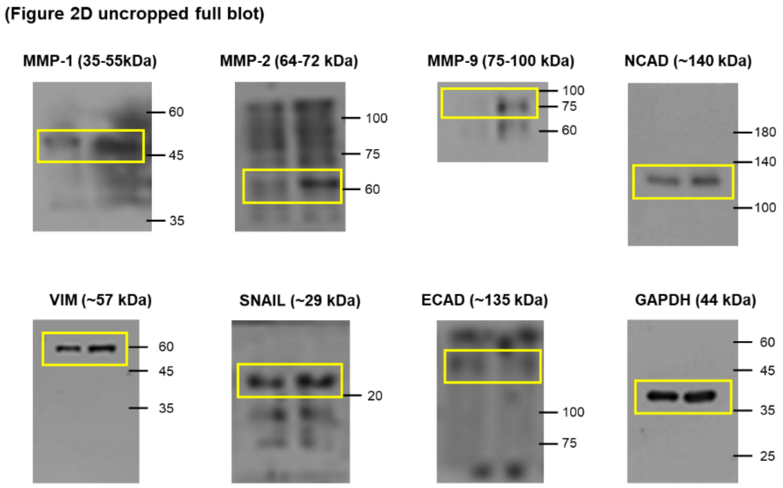

Supplementary Figure S6. Uncropped full blot of western blotting.

Supplementary Table 1. DAVID analysis of sequential gemcitabine-resistant cancer (GRC) cell line from Figure 3B.

Group	Category	Term	Count	%	*P* Value	Genes	List Total	Pop Hits	Pop Total	Fold Enrichment	FDR
1	UP_KEYWORDS	Developmental protein	64	7.5	4.E-05	SEMA5A, FOXA1, TENM4, PLEKHB1, TNFAIP2, HOXA13, CHRDL1, LFNG, HHEX, SHH, MDK, DPYSL2, ZC3H12A, FAM65B, EPHB2, EDIL3, PDGFRB, EDARADD, METRN, WNT5A, SHROOM3, PAX6, PAX5, EREG, KIAA1217, TMEM2, HOXB9, PAX8, ELF3, TRNP1, PRR15, HOXB8, MCTP2, HOXB7, HOXB6, HOXB5, ASF1B, CATSPER1, SEMA7A, DLX3, PDGFB, HOXB13, ADGRG1, RELN, PDPN, MOV10L1, DISC1, PAK3, HOXC6, NTNG1, CHURC1, NPM2, CADM1, UTP3, EYA2, WNT7B, FUZ, BICC1, FMNL3, BMP4, BMP2, FAT3, NES, LGR4	813	949	20581	1.7	2.E-03
1	GOTERM_BP_DIRECT	GO:0008285~negative regulation of cell proliferation	40	4.7	1.E-06	KANK2, IFITM1, ATP8A2, TGFB1I1, PTPRJ, GATA3, PKD2, CDH5, CYP27B1, ADGRG1, RASSF5, RPS6KA2, FTH1, ADORA1, ZNF503, IGFBP7, SOX7, GDF11, TGFB2, RARRES3, TNFRSF9, HMGA1, FUZ, LDOC1, SMARCA2, PTPN14, PML, EREG, BMP4, IL1A, BMP2, IRF1, DLC1, IL1B, CDH13, PTPN6, IRF6, PTGES, DFNA5, MXD4	718	396	16792	2.4	2.E-03
1	GOTERM_BP_DIRECT	GO:0051607~defense response to virus	21	2.5	3.E-05	IFITM3, IFITM1, CD40, IFITM2, APOBEC3G, MX2, MX1, SAMHD1, UNC13D, IFI44L, PML, BST2, PYCARD, MAVS, IFI16, OAS1, OAS2, IRF1, ZC3H12A, F2RL1, TRIM22	718	165	16792	3.0	1.E-02
2	KEGG_PATHWAY	hsa04630: Jak-STAT signaling pathway	16	1.9	2.E-03	IFNB1, IL10RB, IL10RA, IL24, LIF, IL2RG, CSF2RA, IL22RA1, IL6, IFNL2, IFNL1, IL23A, PIM1, IL12A, IL13RA2, IFNL3	306	145	6879	2.5	1.E-01
2	KEGG_PATHWAY	hsa04151: PI3K-Akt signaling pathway	27	3.3	5.E-03	TNXB, ITGB4, LAMA1, LPAR3, LAMC2, FGF1, IL2RG, FGF2, HSP90B1, GNG7, MCL1, NGFR, IFNB1, INSR, NGF, GNG11, COL3A1, IL6, CDK6, COL5A1, PPP2R2C, DDIT4, ITGA6, SGK1, FGFR3, EPHA2, CREB5	306	345	6879	1.8	3.E-01
2	KEGG_PATHWAY	hsa04550: Signaling pathways regulating pluripotency of stem cells	14	1.7	9.E-03	ACVR1, WNT10B, FZD4, LIF, FZD8, SMAD9, WNT9A, KLF4, FGF2, ID1, HESX1, ID3, PCGF1, FGFR3	306	140	6879	2.2	3.E-01
3	KEGG_PATHWAY	hsa04668: TNF signaling pathway	17	2.4	4.E-06	MAP2K3, EDN1, CCL20, MMP3, TNFAIP3, CXCL1, CFLAR, TRAF1, FOS, CXCL3, CXCL2, MMP9, CXCL10, IL6, IL1B, CCL5, JUNB	275	107	6879	4.0	5.E-04
3	GOTERM_BP_DIRECT	GO:0030335~positive regulation of cell migration	19	2.7	8.E-05	RET, CD274, TCAF2, EDN1, SEMA3D, FOXF1, INSR, LAMC2, IRS2, SEMA3F, FGF1, F3, CYR61, CCL5, S1PR1, HAS2, ITGA6, ITGA5, HBEGF	589	184	16792	2.9	3.E-02
3	KEGG_PATHWAY	hsa04630: Jak-STAT signaling pathway	15	2.2	2.E-03	IL11, IFNB1, IL10RA, IL24, LIFR, IL2RG, CSF2RA, CCND3, IL6, IFNL1, IL23A, IL12A, IL13RA2, IL7R, CRLF2	275	145	6879	2.6	6.E-02
3	KEGG_PATHWAY	hsa04151: PI3K-Akt signaling pathway	25	3.6	5.E-03	LAMA5, LAMA1, LPAR1, LAMC2, FGF1, IL2RG, FGF2, CCND3, IFNB1, ITGA2, INSR, VEGFC, NGF, KITLG, IL6, CDK6, PPP2R2C, CCNE1, COL6A1, COL4A6, ITGA6, ITGA5, SGK1, IL7R, EPHA2	275	345	6879	1.8	1.E-01
3	GOTERM_BP_DIRECT	GO:0007050~cell cycle arrest	14	2.0	1.E-03	RB1, CDKN2D, PPP1R15A, CDKN2C, CXCL8, GADD45A, PKD1, ERN1, CUL4A, CDK6, DDIT3, RRAGD, IL12A, GAS6	589	141	16792	2.8	1.E-01
3	KEGG_PATHWAY	hsa04066: HIF-1 signaling pathway	10	1.4	1.E-02	IL6, EDN1, PFKFB3, INSR, SERPINE1, SLC2A1, PLCG2, ENO2, HK2, PDK1	275	96	6879	2.6	2.E-01
3	KEGG_PATHWAY	hsa04390: Hippo signaling pathway	13	1.9	2.E-02	SERPINE1, FZD8, WNT7A, WNT9A, FGF1, WTIP, BMP6, CTGF, CCND3, LATS2, PPP2R2C, ID1, AMH	275	151	6879	2.2	2.E-01
4	KEGG_PATHWAY	hsa01040: Biosynthesis of unsaturated fatty acids	9	0.9	2.E-05	FADS2, SCD, TECR, SCD5, ACOT2, ACOT1, ELOVL6, FADS1, ACOT4	379	23	6879	7.1	2.E-03
5	KEGG_PATHWAY	hsa04142: Lysosome	19	2.5	8.E-06	SCARB2, LAPTM5, CTNS, CTSW, CTSS, CLN5, LAPTM4A, NPC1, GM2A, LAMP1, CTSL, SMPD1, LAMP2, NEU1, SLC17A5, TPP1, ARSG, PPT2, CTSD	316	121	6879	3.4	2.E-03
5	KEGG_PATHWAY	hsa04141: Protein processing in endoplasmic reticulum	18	2.4	2.E-03	PPP1R15A, ERO1B, HSPA5, DERL3, EDEM1, SEL1L, PDIA6, PDIA4, HERPUD1, HSP90B1, SEC61A2, DNAJC3, LMAN1, DDIT3, SEC61G, CANX, P4HB, UGGT2	316	169	6879	2.3	9.E-02

5	KEGG_PATHWAY	hsa04151: PI3K-Akt signaling pathway	31	4.1	5.E-04	LAMA5, TNXB, ITGB4, LAMA1, LAMA4, LPAR3, LAMC2, FGF1, FGF2, HSP90B1, GYS1, ANGPT1, IFNB1, ITGA2, INSR, VEGFC, GNG11, PGF, CDK6, COL4A2, COL4A1, CCNE1, LPAR6, COL6A2, COL6A1, DDIT4, COL6A3, ITGA6, ITGA5, SGK1, IL7R	316	345	6879	2.0	4.E-02
5	GOTERM_BP_DIRECT	GO:0030335~positive regulation of cell migration	19	2.5	3.E-04	ACVR1, TCAF2, HSPA5, SEMA3D, FOXF1, INSR, LAMC2, IRS2, FGF1, MYLK, DAB2, MMP14, PODXL, CCL5, SNAI2, ITGA6, ITGA5, HBEGF, FERMT3	650	184	16792	2.7	1.E-01
6	KEGG_PATHWAY	hsa04015: Rap1 signaling pathway	20	2.7	9.E-04	PDGFRB, ITGB3, PDGFB, LPAR2, PIK3R3, ADCY1, SIPA1L2, RASGRP2, THBS1, IGF1R, FGF5, EFNA1, PARD6B, PARD6A, PLCB4, RASSF5, PDGFD, PLCB1, RAPGEF5, MAP2K6	283	210	6879	2.3	6.E-02
6	KEGG_PATHWAY	hsa01040: Biosynthesis of unsaturated fatty acids	6	0.8	2.E-03	FADS2, SCD, ACOT2, ACOT1, FADS1, ACOT4	283	23	6879	6.3	8.E-02
7	KEGG_PATHWAY	hsa04014: Ras signaling pathway	44	1.3	7.E-02	FOXO4, PLA2G6, RASGRP2, FGF2, RASGRP1, ETS2, IGF1R, PLD2, GNGT1, MAPK8, HTR7, SYNGAP1, RRAS, GNG4, GNG7, PDGFD, PLCG2, ABL1, ABL2, RAC3, PLCE1, MAPK3, PAK4, PRKCG, NGFR, ANGPT4, MAP2K2, INSR, PLA2G4C, PLA2G4A, KSR2, NGF, GNG11, GRIN1, EFNA3, MRAS, RASA1, JMJD7-PLA2G4B, FGFR4, FGFR3, RAB5A, FGFR2, EPHA2, ARF6	1052	226	6879	1.3	1.E+00
7	KEGG_PATHWAY	hsa04010: MAPK signaling pathway	65	2.0	2.E-05	ATF2, HSPB1, ARRB2, ECSIT, DUSP16, FGF2, RPS6KA6, PPP3CC, MYC, RAC3, MAP3K5, PRKCG, MAP2K3, DUSP5, DUSP2, MAP2K2, DUSP1, IL1R1, CACNA2D1, PLA2G4C, CACNA2D2, PLA2G4A, FOS, DUSP8, NGF, MAPK8IP2, DUSP9, DUSP6, DUSP7, CACNB4, MRAS, MAPKAPK3, RASA1, JMJD7-PLA2G4B, DDIT3, MAPT, RASGRP2, RASGRP1, CACNA1G, RELB, CACNG6, CACNG7, CACNG8, MAPK8, RRAS, FLNB, MAP2K5, MAPK3, MAP4K4, NTRK1, JUN, JUND, GADD45B, TGFB3, GADD45A, BDNF, NFATC1, NFKB2, NR4A1, PPP5C, MAP3K13, FGFR4, FGFR3, FGFR2, MAP3K12	1052	253	6879	1.7	5.E-03
8	KEGG_PATHWAY	hsa05202: Transcriptional misregulation in cancer	33	1.2	6.E-02	DDX5, CD40, CSF2, LDB1, HDAC1, TMPRSS2, PLAT, FOXO1, AFF1, HOXA10, LYL1, HHEX, FUT8, RXRA, CD14, HIST1H3E, MEN1, JUP, FUS, LMO2, HMGA2, PAX5, FLI1, NFKB1, PML, ETV6, ETV7, SPINT1, PAX8, EWSR1, PPARG, MET, CDK14	1005	167	6879	1.4	2.E-01
8	GOTERM_BP_DIRECT	GO:0016477~cell migration	42	1.5	3.E-04	CD63, SDC4, BRAT1, ITGB3, SDC3, ADAMTS12, THBS1, ADGRG1, FUT8, STRIP2, CDH2, PSTPIP2, PODXL, SIX2, NCK2, CSK, PLCG1, TSPAN1, TNS3, HOXA5, RHBDF1, NANOS1, TGFB2, ZRANB1, JUP, STYK1, PTK6, ARPC5, PARP9, DGKZ, VAV2, FMNL3, TGFBR3, TIAM1, TAOK2, ELMO2, PLXNB1, CSPG4, LCP1, DOCK1, NOX1, BCAR1	2327	172	16792	1.8	9.E-02

Supplementary Table 2. Biological characteristics and coefficients values of 23-genes signature from Cox linear regression analysis.

N.	Gene ID	Biological characteristics (Phase)	Coefficient	*P* value
1	*GATA3*	Development (P0)	-1.34	< 0.001
2	*FOXA1*	Development (P0)	-0.82	< 0.001
3	*RASSF5*	Development (P0)	-0.48	< 0.001
4	*PTPN6*	Development (P0)	-0.78	< 0.001
5	*TRNP1*	Development (P0)	-1.00	< 0.001
7	*APOBEC3D*	Defense response to virus (P0)	-0.23	< 0.001
6	*APOBEC3G*	Defense response to virus (P0)	-2.07	< 0.001
8	*ABL1*	RAS signaling pathway (P15)	0.32	< 0.001
9	*ABL2*	RAS signaling pathway (P15)	0.22	< 0.001
10	*CACNA2D1*	MAPK signaling pathway (P15)	0.56	< 0.001
11	*JUN*	MAPK signaling pathway (P15)	0.16	< 0.001
12	*GNG4*	MAPK signaling pathway (P15)	0.57	< 0.001
13	*NGF*	MAPK signaling pathway (P15)	0.64	< 0.001
14	*MYC*	MAPK signaling pathway (P15)	0.72	< 0.001
15	*MAPK4K4*	MAPK signaling pathway (P15)	0.26	< 0.001
16	*STC1*	Secreted (P15)	1.49	< 0.001
17	*FOXD1*	EMT-related (P15)	1.30	< 0.001
18	*FOXC2*	EMT-related (P15)	0.34	< 0.001
19	*TGFB1*	TGFβ signaling pathway (P15)	0.20	< 0.001
20	*TGFB3*	TGFβ signaling pathway (P15)	0.32	< 0.001
21	*NOG*	TGFβ signaling pathway (P15)	0.39	< 0.001
22	*SMAD9*	TGFβ signaling pathway (P15)	0.81	< 0.001
23	*NT5E*	Gemcitabine mechanism of action (P15)	0.69	< 0.001

Supplementary Table 3. qRT-PCR primer sequence list

Gene	Forward Primer Sequences	Reverse Primer Sequences
GAPDH	TGCACCACCAACTGCTTAGC	GGCATGGACTGTGGTCATGAG
MMP-1	TTTGGCTTCCCTAGAACTGTG	GCTATCATTTTGGGATAACCTGG
MMP-2	GCGGCGGTCACAGCTACTT	CACGCTCTTCAGACTTTGGTTCT
MMP-9	CCTGGAGACCTGAGAACCAATC	CCACCCGAGTGTAACCATAGC
VIM	AGGCAAAGCAGGAGTCCACTGA	ATCTGGCGTTCCAGGGACTCAT
SNAIL	CCACAAGCACCAAGAGTC	TGGCAGTGAGAAGGATGT
SLUG	TTCACTCCGAAGCCAAATG	TCTCTCTGTGGGTGTGTG
ZEB1	TGTGCCAATTTGTTCCTGTA	TGAGATGGGAGTCTGGTAAA
ZEB2	ATCGTGTAACAAAGATGAAGAAA	TCACAAATGTCTCAAGTTCTAAA
TWIST	GCCAGGTACATCGACTTCCTCT	TCCATCCTCCAGACCGAGAAGG
NCAD	GAATTCAGCACCCCCCTCAG	GCTGCATATATCGATCTGGG
SDC1	TTCACACTCCCCACACAGAG	ACTACAGCCGTATTCTCCCC
SDC2	TGTACCTTGACAACAGCTCC	CTCTACATCCTCATCAGCTCC
ECAD	GCAGTGACGAATGTGGTACC	GTGTCTGGCTCCTGGGCAGT
FOXC2	ATGTTCGAGAACGGCAGCTT	CTTCTTCTCGGCCTCCTTGG
FOXD1	CTCGTATATCGCGCTCATCA	TCTTGACGAAGCAGTCGTTG
GNG4	ACCACTAGCATCTCCCAAGC	CAGGCACTGGAATGATGAGA
NGF	GGGGCATTGACTCAAAGCAC	TTCCTGCTGAGCACACACAC
NOG	CCAGCACTATCTCCACATCC	GTCTCGTTCAGATCCTTTTCC
NT5E	CCCATTCTTCTAAACAGCAGCATTC	CTAAAGCGGCATGATTGAGAGG
SMAD9	CCACAGAAGCCTCTGAGACC	CCCAACTCGGTTGTTCAGTT
STC1	AGCGCTGCTAAATTTGACACT	CTTTGGAAAGTGGAGCACCTCCG
APOBEC3D	TGCAGAAAGGTGCTTCCTCT	GCGGTGAAGATGGTGAGATT
APOBEC3G	TTCAGCACTGTTGGAGCAAG	TGAAAGTGAATGTGGGTGGA
IFNB1	CTTGGATTCCTACAAAGAAGCAGC	TCCTCCTTCTGGAACTGCTGCA
IL10RA	GCCGAAAGAAGCTACCCAGTGT	GGTCCAAGTTCTTCAGCTCTGG
IL23A	GAGCCTTCTCTGCTCCCTGATA	GACTGAGGCTTGGAATCTGCTG
IL6	AGACAGCCACTCACCTCTTCAG	TTCTGCCAGTGCCTCTTTGCTG
IL2RG	CACTCTGTGGAAGTGCTCAGCA	GAGCCAACAGAGATAACCACGG
CTSW	CGTGACCATCAACATGAAGCCC	CCTCTGACTTGACGCTGCCAAA
LAPTM5	TTCCATCGCCTTCATCACTGTCC	CTCTTCTCCTCCACCGAGTTCA
CLN5	CTGTCCAACTGGCTCACCTATC	ACTTCTGAATCCAATGGCATCATG
GM2A	TACCTATGGGCTTCCTTGCCAC	GACGCTCTCTATGCGGTAGTTC
SCARB2	GCCAATACGTCAGACAATGCCG	CTCATCTGCTTGGTAAAAGTGTGG
CACNA1G	TTCACCGCAGTCTTTCTGGCTG	TGACGGAGATGAGCACCAACAG
MYC	CCTGGTGCTCCATGAGGAGAC	CAGACTCTGACCTTTTGCCAGG
RRAS	ACGAAGATCTGCAGTGTGGA	TTAATGGCGAACACCAGCAG
JUN	AGCCCAAACTAACCTCACG	TGCTCTGTTTCAGGATCTTGG
JUND	TCTCTCAAGCTCGCCTCTTC	GGGGTAAAAGTACTGTCCCG
